# The Role of Pharmacometrics in Advancing the Therapies for Autoimmune Diseases

**DOI:** 10.3390/pharmaceutics16121559

**Published:** 2024-12-05

**Authors:** Artur Świerczek, Dominika Batko, Elżbieta Wyska

**Affiliations:** Department of Pharmacokinetics and Physical Pharmacy, Faculty of Pharmacy, Jagiellonian University Medical College, 9 Medyczna Street, 30-688 Krakow, Poland; dominika.batko@student.uj.edu.pl (D.B.); e.wyska@uj.edu.pl (E.W.)

**Keywords:** pharmacometrics, autoimmune diseases, personalized medicine, PBPK modeling, PK/PD modeling, disease progression modeling, population modeling, AI in drug development

## Abstract

Autoimmune diseases (AIDs) are a group of disorders in which the immune system attacks the body’s own tissues, leading to chronic inflammation and organ damage. These diseases are difficult to treat due to variability in drug PK among individuals, patient responses to treatment, and the side effects of long-term immunosuppressive therapies. In recent years, pharmacometrics has emerged as a critical tool in drug discovery and development (DDD) and precision medicine. The aim of this review is to explore the diverse roles that pharmacometrics has played in addressing the challenges associated with DDD and personalized therapies in the treatment of AIDs. **Methods**: This review synthesizes research from the past two decades on pharmacometric methodologies, including Physiologically Based Pharmacokinetic (PBPK) modeling, Pharmacokinetic/Pharmacodynamic (PK/PD) modeling, disease progression (DisP) modeling, population modeling, model-based meta-analysis (MBMA), and Quantitative Systems Pharmacology (QSP). The incorporation of artificial intelligence (AI) and machine learning (ML) into pharmacometrics is also discussed. **Results**: Pharmacometrics has demonstrated significant potential in optimizing dosing regimens, improving drug safety, and predicting patient-specific responses in AIDs. PBPK and PK/PD models have been instrumental in personalizing treatments, while DisP and QSP models provide insights into disease evolution and pathophysiological mechanisms in AIDs. AI/ML implementation has further enhanced the precision of these models. **Conclusions**: Pharmacometrics plays a crucial role in bridging pre-clinical findings and clinical applications, driving more personalized and effective treatments for AIDs. Its integration into DDD and translational science, in combination with AI and ML algorithms, holds promise for advancing therapeutic strategies and improving autoimmune patients’ outcomes.

## 1. Introduction

Autoimmune diseases (AIDs) encompass a broad spectrum of disorders characterized by the aberrant attack of the immune system on the body’s own tissues. These diseases, including rheumatoid arthritis (RA), multiple sclerosis (MS), systemic lupus erythematosus (SLE), Sjögren’s syndrome (SjS), systemic sclerosis (SS), inflammatory bowel diseases such as Crohn’s disease (CD) or ulcerative colitis (UC), and autoimmune liver diseases such as autoimmune hepatitis (AIH), primary sclerosing cholangitis (PSC), and primary biliary cholangitis (PBC), as well as various types of myositis, present unique challenges in treatment due to their complex and often poorly understood pathophysiology, limited response to existing treatments, and numerous side effects related to immunosuppressive medications [1]. The variability in disease presentation and progression (DisP), combined with significant differences in how individual patients respond to therapies, complicates the development of universally effective treatments [2]. Furthermore, the drugs used to manage these conditions often come with significant side effects and long-term safety concerns, particularly because many of these treatments involve immunosuppression, which can increase the risk of infections and malignancies. The unpredictable nature of AIDs adds another layer of difficulty, making it challenging to assess the long-term efficacy of treatments, while the high costs and significant risks associated with drug research and development in this area further complicate efforts to bring new therapies to market.

One of the critical challenges in treating some AIDs is a limited number of specific biomarkers that can be used to accurately assess disease activity and progression, and to predict patients’ response to treatments [3]. This makes it difficult to tailor treatments to individual patients, often leading to suboptimal dosing and an increased risk of flares and treatment failures, or creating the potential for adverse drug reactions. Moreover, the complexity of treatment regimens for autoimmune patients raises the risk of drug–drug interactions, particularly as these patients often require long-term and multi-drug therapy to manage their condition [4]. The identification and measurement of appropriate clinical endpoints for AIDs can also be challenging, further complicating efforts to evaluate the effectiveness of new therapies.

The access to innovative treatments involving biologic drugs, due to high costs, is substantially limited in low- and middle-income countries, which further negatively impacts the outcomes of treatments for AIDs in underdeveloped and developing nations [5]. These challenges highlight the need for innovative approaches that can enhance our understanding of AIDs, improve the efficacy of treatment regimens, and ultimately lead to the development of safer, personalized, and more accessible and effective therapeutic options.

Pharmacometrics offers a potential solution to address these challenges by providing a set of quantitative tools that can facilitate new drug discovery and development and optimize dosing strategies for AIDs. In this review, we focus on pharmacometric techniques such as Physiologically Based Pharmacokinetic (PBPK) modeling [6], Pharmacokinetic/Pharmacodynamic (PK/PD) modeling [7], DisP modeling [8], Quantitative Systems Pharmacology (QSP) [9], Boolean networks [10], model-based meta-analysis (MBMA) [11], non-linear mixed-effect (NLME) (commonly known as population (Pop)) modeling, and Bayesian hierarchical modeling, which have shown significant potential in this regard [12,13].

PK/PD models provide a detailed understanding of the relationship between drug concentrations at the site of action and their pharmacological effects. DisP models assume that diseases follow quantifiable patterns over time, which can be mathematically characterized. In the context of AIDs, these models are useful in predicting disease evolution and assessing the impact of various therapeutic interventions. DisP and QSP models can help to identify critical disease biomarkers and enhance our understanding of long-term treatment effects. These models are especially useful in diseases such as MS, where the course of the disease can be highly variable and difficult to predict. QSP models take a holistic approach by integrating drug action with disease biology, enabling a deeper understanding of the complex pathophysiological mechanisms underlying autoimmune conditions. QSP models can simulate the effects of medications on various biological pathways, helping to identify novel therapeutic targets and predict the outcomes of different treatment strategies. By providing a framework for integrating data from multiple sources, QSP and Boolean network models can help to unravel the signaling pathways that drive DisP, leading to the development of more effective and targeted therapies. NLME and Bayesian hierarchical models are used to quantify different layers of variability in drug PK and response, as well as DisP, which is a common challenge in autoimmune therapies. By incorporating patient-specific factors into these models, referred to as covariates, it is possible to predict the PK, PD, and progression of AIDs, allowing for more personalized treatment strategies. This is particularly important in AIDs, where the therapeutic window of immunosuppressive drugs can be narrow, and the consequences of under- or overdosing can be severe.

The application of these approaches in clinical settings marks a significant shift towards more personalized, predictive, and precise treatment strategies for AIDs, leading to a more rational use of medications and potentially reducing costs associated with treatment failures or adverse reactions. Finally, the use of pharmacometrics in drug discovery and development can help to reduce the costs and risks associated with bringing new therapies to market, by providing more accurate and efficient means of assessing the safety and efficacy of new drugs.

In addition to the traditional pharmacometric approaches, the incorporation of artificial intelligence (AI) and machine learning (ML) into pharmacometrics shows significant potential for further refining these models and enhancing their predictive performance. AI and ML techniques are capable of handling large datasets to identify patterns and relationships that may not be apparent using traditional statistical methods. By integrating AI and ML with pharmacometrics tools, it is possible to create more robust and accurate models that can better predict patient outcomes and guide the development of new therapies.

The literature search was conducted in the PubMed database, using specific combinations of keywords to retrieve relevant studies. The keywords consisted of each modeling technique listed in this article, paired with each of the AIDs mentioned in this review. For example, combinations like “PBPK modeling and Rheumatoid Arthritis”, “PK/PD modeling and Multiple Sclerosis”, “Disease Progression modeling and Type 1 Diabetes”, and similar pairs were utilized to capture a comprehensive scope of studies applying these techniques in the context of AIDs. In addition, combinations of the keywords “machine learning” and “artificial intelligence” with the names of the AIDs were also used to identify relevant studies in the PubMed database.

The aim of this review article is to explore the diverse roles that pharmacometrics has played in the past 20 years in addressing the challenges associated with the discovery and development of new drugs for AIDs and delivering personalized therapies for patients with these diseases. This review seeks to highlight the significant strides made in enhancing our understanding of AIDs and the therapeutic interventions used to manage them. It aims to provide a comprehensive synthesis of the current state of research, showcasing how pharmacometrics techniques contribute to the optimization and personalization of treatment regimens and the development of new treatment strategies. This includes an examination of how these methods address patient-specific variables and disease heterogeneity, improve the safety and efficacy of drug dosing, and ultimately guide the development of more effective and safer therapeutic options. Additionally, this review will touch upon the emerging role of AI and ML in complementing pharmacometric approaches, providing insights into how their integration can further refine the outcomes of modeling in AID treatment. Through this review, we aim to present a detailed perspective on the transformative impact of pharmacometrics on the treatment of AIDs, offering insights into future directions and potential advancements in this area.

## 2. Overview of Autoimmune Diseases

Humans possess immunological tolerance acquired throughout life, which enables the immune system to distinguish between self- and non-self antigens. However, dysregulation of this mechanism can result in autoimmunity, where the immune system erroneously recognizes self-proteins as foreign, leading to an immune response against the body’s own tissues. The process of autoimmunization can be triggered by various factors, though its precise etiology remains unknown. It may stem from incomplete elimination of self-reactive lymphocytes in the thymus during clonal anergy, clonal deletion, or at the stage of secondary selection. It is important to note that the presence of autoreactive T and B lymphocytes in the body does not necessarily indicate a pathological state or the development of an AID. These cells can be present in healthy individuals, and they play a role in maintaining homeostasis by removing degraded self-cells. The genetic basis of autoimmunity includes certain rare monogenic autoimmune disorders, such as autoimmune polyendocrinopathy syndrome type 1 (APS1), or IL-2Rα deficiency. Although the majority of AIDs are not hereditary, some genetic factors may predispose individuals to the development of autoimmunity. Environmental factors also have a significant impact on the onset of AIDs; these include heavy metals, tobacco smoke, medications, food components, and infectious agents. Regarding the latter, molecular mimicry may occur, where the antigens of pathogens closely resemble self-antigens, leading the immune system to target the body’s own tissues instead of the pathogens [14,15].

**Figure 1:**
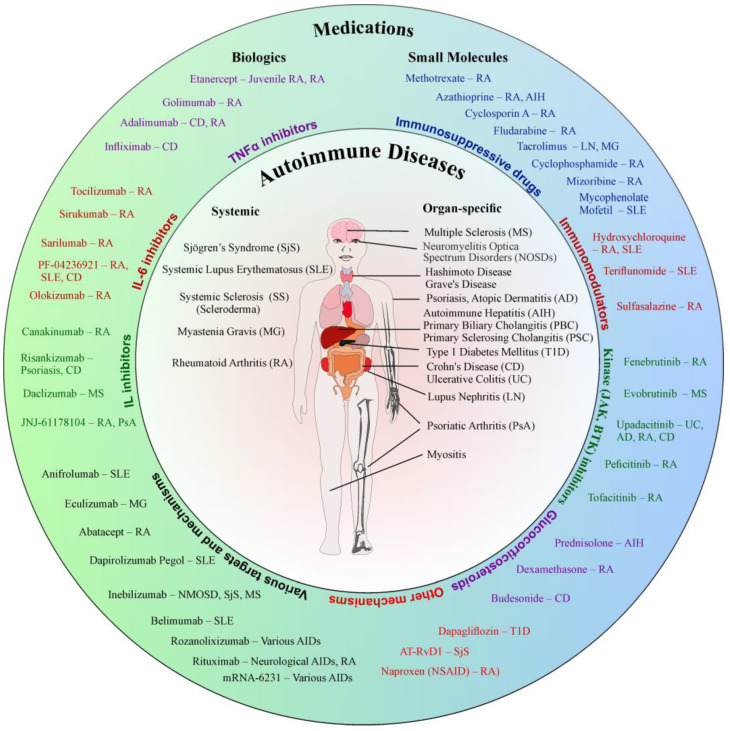
Schematic representation of systemic and organ-specific AIDs, and example medications used in the treatment of these conditions.

Statistics indicate that one in ten individuals suffers from an AID [16]. These conditions encompass a broad spectrum of disorders that can be either organ-specific or systemic (Figure 1). Diseases with disseminated foci throughout the body include SLE, RA, SS, SjS, and MG. SLE is a connective tissue disorder characterized by a diverse clinical course, affecting multiple organs. A study conducted in 2022 estimated that the global incidence rate was 5.14 per 100 thousand person-years. Diagnosis of the disease requires that the patient meets the 2019 EULAR/ACR classification criteria, which include, among others, the antinuclear antibody (ANA) test, the anti-dsDNA test, and the occurrence of symptoms [17]. RA is one of the most commonly observed chronic AIDs, primarily affecting the joints. This condition often causes pain, stiffness, limited mobility, and joint swelling. It can also lead to the development of rheumatoid nodules, lung involvement, or vasculitis, and it may accelerate the progression of atherosclerosis [18]. The diagnosis of RA is conducted in patients who report joint pain and swelling, with laboratory results indicating abnormalities such as elevated CRP levels. The 2010 EULAR/ACR classification criteria are used for diagnosis, taking into account the number of affected joints, duration of symptoms, serological changes, and CRP levels [18]. SjS is a systemic disease characterized by dysfunction of the lacrimal and salivary glands, leading to dryness of the eyes and mouth. It can also manifest with a variety of symptoms affecting nearly every organ, with the range and nature of these symptoms varying between patients [19]. According to a 2015 meta-analysis, the incidence of SjS is 6.92 per 1 million person-years [20]. The disease is diagnosed based on the 2016 EULAR/ACR classification criteria in patients who have experienced dryness for more than three months. These criteria include tests for the presence of SjS-A/Ro antibodies [21]. SS is an autoimmune connective tissue disease characterized by progressive fibrosis of the internal organs and skin. Patients often present with Raynaud’s phenomenon (vascular changes) and fatigue prior to the onset of full-blown disease. The diagnosis of SS is based on the 2013 EULAR/ACR classification criteria, which include the presence of anti-Scl-70 antibodies and the assessment of skin thickness [22].

Organ-specific AIDs are conditions in which the inflammatory process is limited to a specific area. These diseases encompass a diverse group of disorders that can affect organs such as the thyroid, pancreas, and hematopoietic system. Examples of organ-specific AIDs include Hashimoto’s disease, Graves’ disease, type 1 diabetes (T1D), MS, and AIH. Some of the most common AIDs are thyroid disorders. Hashimoto’s disease is a chronic lymphocytic thyroiditis characterized by the production of antibodies against thyroglobulin and thyroid peroxidase. This disease primarily affects women and leads to progressive thyroid fibrosis, with some cases resulting in hypothyroidism. Patients with Hashimoto’s disease typically present with elevated TSH levels, normal or low thyroxine levels, dry and cold skin, bradycardia, facial swelling, and significant fatigue. Diagnostic evaluation may also include measuring anti-TPO antibody levels. The incidence of this disease is 350 per 100 thousand women per year and 80 per 100 thousand men per year [23]. The second thyroid-related disease is Graves’ disease. In this condition, B lymphocytes synthesize thyroid-stimulating immunoglobulins (TSIs), leading to thyroid enlargement and increased synthesis of thyroid hormones. Symptoms of Graves’ disease may include goiter, heart palpitations, irritability, and excessive sweating. Biochemical tests typically show low TSH levels with elevated thyroxine and triiodothyronine levels. For more accurate diagnosis, measuring TRAb antibody levels is also recommended [24].

T1D is an AID characterized by the destruction of pancreatic β-cells. There is a genetic predisposition associated with the HLA system, which increases the risk of developing the disease [25]. According to data from the International Diabetes Federation, in 2021, 31 new cases of T1D per 1000 children aged 0 to 19 years were diagnosed in Europe [26]. Children suspected of having this disease typically present with symptoms such as increased thirst, polyuria, drowsiness, and weight loss. The primary diagnostic parameter for T1D is blood glucose concentration [25].

MS is a chronic inflammatory disease characterized by widespread demyelination of the brain and spinal cord. This can lead to symptoms such as vision impairment, balance problems, weakness, and numbness in the limbs [27]. According to the Multiple Sclerosis Atlas, in 2020, the prevalence of the disease was 35.9 per 100 thousand people [28]. The diagnosis of MS is based on the 2017 McDonald criteria, which allow for the assessment of the disease type based on the number of relapses and clinical symptoms [29].

AIDs most commonly affect young individuals, who require specialized testing, continuous pharmacological treatment, and regular care from a specialist to maintain an appropriate quality of life. Daily activities often necessitate support from family members. All of these aspects are costly, and in some cases, access to them is limited, especially in low- and middle-income countries. There is a constant need for new therapies because many patients do not respond to the available medications. For instance, in Poland, a middle-income EU country with a population exceeding 37.6 million people, treatment for most AIDs involves the use of non-steroidal anti-inflammatory drugs (NSAIDs), corticosteroids, and immunosuppressants.

However, the use of biologic drugs requires meeting specific criteria and enrolling in a drug program. According to the report titled “Valuation of Benefits in Drug Programs as a Key Element in Developing Strategies to Increase Access to Innovative Therapies in Autoimmune Diseases”, in 2018, the National Health Fund (NFZ) allocated over 63.3 million EUR and more than 16 million EUR for drug reimbursements in two drug programs for MS. Additionally, more than 6.3 million EUR and over 0.9 million EUR were spent on healthcare services, respectively.

In addition to the direct costs of reimbursements and healthcare services, the report also considers indirect costs associated with AIDs, primarily including sickness absenteeism and disability pensions due to partial or total incapacity to work. In 2010, the Social Insurance Institution (ZUS) paid out benefits amounting to 49.5 million EUR to individuals with RA. From 2012 to 2015, the annual costs generated by Polish patients with MS ranged from 42.7 million EUR to 116.2 million EUR, with the highest burdens related to patients whose therapy was ineffective and who experienced exacerbations more frequently than remissions. The data presented above refer to the funds paid out by the Polish NFZ and ZUS. In high-income countries, these costs for healthcare systems are probably even higher.

Moreover, patients also bear significant costs out of their own pockets, such as expenses for over-the-counter (OTC) and prescription medications, private medical consultations, and diagnostic tests. It should also be stressed that the access to drug programs is often limited in numbers (for example, to about 20% of AID patients in Poland) and duration, forcing patients to seek additional services. Additionally, the families of patients often need to take leave from work or even resign from employment to provide care for their loved ones [30].

The treatment of AIDs usually lasts a lifetime, as these conditions are incurable. Especially with such long-term therapy, it is important to monitor the patient for side effects and assess the risk–benefit ratio of the treatment. The most common side effects include nausea, weakened immunity, and drowsiness, but some medications have more specific and severe adverse effects. The primary and most frequently used drugs are glucocorticoids, which are used in conditions such as RA, SLE, AIH, and vasculitis. They can cause hypertension, heart rhythm disorders, abnormal blood glucose levels, and osteoporosis. Additionally, they increase the susceptibility to bacterial and fungal infections. Other drugs used in the treatment of AIDs include cyclophosphamide, an alkylating agent used in SLE, which can cause suppression of bone marrow function and hemorrhagic cystitis, as well as increasing the susceptibility to infections. Methotrexate, used in the treatment of RA, is a folic acid antagonist and may cause hepatotoxicity and toxic effects on bone marrow. Azathioprine, an immunosuppressive drug used in conditions such as SLE, AIH, and RA, is hepatotoxic and increases the risk of malignancies.

In the case of AIDs, biologic drugs are also often used, which are characterized by a lower number of side effects in comparison to the small-molecule drugs, although they can still occur. For example, biologics used for the treatment of AIDs, such as infliximab, anakinra, belimumab, and adalimumab, may increase the risk of bacterial infections. Moreover, the administration of biologic agents may lead to immunogenicity, characterized by the production of anti-drug antibodies (ADAs) that can negatively impact the therapeutic efficacy [31].

In addition to the aforementioned drugs, patients may receive medications that help manage symptoms, such as insulin for T1D or thyroid hormones for Hashimoto’s disease. These can also affect the patient’s well-being and cause side effects. In AIDs, it is important to regularly monitor the patient’s health and conduct tests to reduce the risk of life-threatening situations [32].

## 3. Biomarkers and Clinical Outcomes in Autoimmune Diseases

Biomarkers allow for the assessment of whether a pathogenic process is present, the monitoring of its activity, and the evaluation of the effectiveness of therapy. Biomarkers should be measurable through qualitative and/or quantitative testing. They are particularly important in AIDs, where diagnosis and monitoring of disease progression can often be challenging due to non-specific symptoms of these disorders. Ideally, a biomarker should be linked to the pathophysiology of the disease, be sensitive and specific, and be easily detectable by established tests. Biomarkers are crucial in the development of PD, PK/PD, QSP, DisP, and Boolean network models, in which they allow for the quantitative assessment of the pharmacological effects of treatments, contributions of specific signaling pathways to clinical outcomes, potential toxic effects of therapeutics, and disease progression. Table 1 presents examples of biomarkers utilized in the diagnosis and monitoring of AIDs [33].

In the monitoring of DisP and treatment effects, clinical outcomes are of critical importance. They offer quantifiable measures that reflect the physiological or pathological state of an individual and their therapeutic response to a treatment. Clinical outcomes are utilized both in clinical practice and in the development of pharmacometric models. These models depend on clinical outcomes to simulate disease dynamics and drug effects within the body. By integrating clinical outcomes into mathematical frameworks, we can enhance the precision and usefulness of predictive modeling in clinical practice.

Both biomarkers and clinical outcomes may be represented by different data types, such as continuous data, used to describe variables like cytokine concentrations or enzyme activity; categorical data, which analyze outcomes, such as responder status or symptom severity; count data, including events such as seizure counts in epilepsy; time-to-event data, including survival models such as Cox proportional hazards, analyzing data on the time until therapeutic failure; repeated-measures data, which are common in longitudinal studies; binary data, such as yes/no outcomes (e.g., success/failure), which are often modeled with logistic regression; and ordinal data, which are ordered clinical outcomes, such as pain scales, modeled using ordinal logistic regression. Examples of clinical outcomes used in the monitoring of AIDs and in mathematical modeling are presented in Table 2.

## 4. A Brief Overview of Basic Pharmacometric Methods

Pharmacometrics is an interdisciplinary field of science that involves the construction of mathematical models to define, challenge, and resolve queries surrounding biological processes. It plays a crucial role in decision-making during drug development and translational research settings. The field has evolved beyond just quantitative analysis methods and is closely related to clinical pharmacology, sharing common research areas and decision-making expectations. It has significant potential to influence drug development and precision medicine, as evidenced by its impact on corporate infrastructures and regulatory review processes over the past 20 years [46].

PBPK modeling is a tool that allows for a mathematical description of how drugs are absorbed, distributed, metabolized, and excreted, based on physiological characteristics of the human or animal body and physicochemical properties of the medication. This approach is currently the most mechanistic among other PK methodologies, such as compartmental modeling and non-compartmental analysis (NCA). The assumption underlying PBPK modeling is that the body can be represented as a series of interconnected compartments, each corresponding to a different organ or tissue, with specific physiological characteristics. Drug concentration–time profiles in these compartments may be described by a set of ordinary differential equations (ODEs) [47]. This approach uses the volumes of organs and tissues, blood flows through these compartments, and tissue-to-plasma partition coefficients of a drug. Today, more complex PBPK models are being developed that describe drug kinetics in different compartments of a given organ, or even at a subcellular level. In AIDs, PBPK modeling is particularly useful for predicting how variations in disease states, such as inflammation, organ impairment, and changes in blood flow through the organs, can affect drug kinetics [48]. It is especially useful in understanding and assessing the impact of drug–drug interactions, which is essential for autoimmune patients, who are often taking multiple medications [49].

Mechanistic PK/PD modeling integrates the PK of a drug (how the body affects a drug) with its PD (how a drug affects the body). This approach typically assumes a direct or indirect cause-and-effect relationship between drug concentration and its pharmacological effect, often quantified using a specific biomarker of pharmacological response or a clinical outcome [7]. The direct-effect PD models include maximum effect (Emax) and maximum inhibition (Imax) models that allow for the capture of non-linear relationships between the drug’s concentration at the site of action and its effect. This effect may constitute an increase or decrease in the quantity of an observed biomarker. There are different types of these models involving Hill’s coefficient, which are referred to as sigmoidal Emax and Imax models [7]. These simple PD models are commonly incorporated in many statistical software packages.

In 1969, Nagashima et al. from the University at Buffalo characterized an indirect response based on the anticoagulant effect of warfarin [50]. Prof. Jusko and coworkers, in the early 1990s, further developed this concept and indicated applications of a family of four indirect-response models in relation to different groups of drugs and their mechanisms of action [51]. Indirect-response models assume a constant production of a biomarker representing a pharmacological response and its first-order loss from the system, leading to the achievement of a steady-state quantity of this biomarker [51]. According to the assumptions of these models, drugs or various signaling molecules may stimulate or inhibit production or loss of the pharmacological response. The indirect-response models have been increasingly used due to their relevance to a broad array of physiological processes. Additionally, they account for a commonly observed delay between drug intervention and the pharmacological effect, which may arise from the indirect mechanism of action of a given drug.

Biophase distribution models, also known as effect-compartment models, belong to the group of direct-response models. They assume that the observed effect of a medication correlates with its concentration at the site of action, which is represented by a hypothetical effect compartment (biophase). These models can capture a delay observed between the maximum concentration of some drugs in the blood and their maximum observed pharmacological response. This delay, according to this model, is caused by the time required for a drug to be distributed from the blood to the site of action [52].

Transit compartment models are used to mathematically represent multistep processes of signal transduction through biological pathways starting from the interaction of a drug with a target (receptor or enzyme), ending up at the observed pharmacological response [53]. Target-mediated drug disposition (TMDD) models are commonly used to describe complex non-linear PK of small molecules and biologic drugs that are affected by the high capacity of a target and the strong affinity of these medications to the target [54]. More complex mechanistic PK/PD models have now been developed, which are constructed using various combinations of indirect-response models and transit compartments, including irreversible binding of a drug to a target [41,55].

QSP modeling is currently the most mechanistic and holistic PD modeling approach, integrating pharmacology with systems biology to describe drug effects and pathological states within the context of biological systems [9]. QSP models typically assume that drug effects can be understood as part of a complex network of biological pathways and processes. For AIDs, QSP can be especially valuable in elucidating the disease’s underlying pathophysiological mechanisms and identifying the most important signaling pathways involved in disease pathogenesis. In addition, this approach aids in the identification of novel therapeutic targets and biomarkers [56,57,58].

Boolean networks are mathematical models that are used to represent and analyze complex systems involving binary states, e.g., “true” or “false”. Each node in a Boolean network represents a variable that can take one of two possible values (0 or 1). The state of each node is determined by a Boolean function of the states of a subset of nodes in the network. The network evolves over discrete timesteps, with the state of each node updating according to its Boolean function [10]. In QSP modeling, Boolean networks play a role in simplifying biological networks, where detailed kinetic parameters may be unknown or difficult to measure or estimate.

DisP modeling is a mathematical approach that is utilized to describe and predict the course of a disease over time. In the context of AIDs, these models integrate various data sources, such as patient medical history, genetic factors, biomarkers, clinical outcomes, and response to previous treatments, to understand how a disease evolves in an individual [59,60,61]. These models may constitute simple empirical algebraic functions, e.g., exponential functions or logistic growth functions, or they may be more mechanistic in their nature, involving transit compartments and feedback mechanisms, biological interactions, and detailed physiological processes represented by the means of ODEs [8]. In pre-clinical settings, DisP models utilize the data from studies in animal models to map the trajectory of the disease, identifying key biological markers and pathways involved in the disease process. This enables researchers to predict how a disease might progress in humans and assess the potential efficacy and safety of new treatments prior to clinical trials [62,63,64,65].

MBMA is an approach that integrates published summary data with internal datasets to support critical decision-making throughout the drug development process. This approach is particularly valuable in evaluating the benefit–risk profile of investigational treatments. MBMA facilitates the identification of optimal dosing strategies by incorporating data from historical external comparators for specific disease indications, including AIDs. Its flexibility in synthesizing and interpreting aggregated data makes it an important component of pharmacometrics, enabling more informed evaluations of therapeutic interventions [11].

Population NLME and Bayesian hierarchical models are advanced pharmacometric tools that account for variability at multiple levels, making them indispensable in PK/PD analyses [12,66]. Population NLME modeling provides a framework to analyze PK/PD data by simultaneously estimating both fixed effects (population-averaged parameters) and random effects (individual-specific deviations from the population mean) [66]. This approach allows for the modeling of inter-individual and intra-individual variability in a comprehensive manner. Bayesian hierarchical models complement this by integrating prior knowledge and enabling full probabilistic inference, offering posterior distributions for both population-level and individual-level parameters [67]. These methods are particularly powerful in handling sparse or unbalanced data, a common scenario in clinical trials, and they facilitate predictions of drug behavior in new populations. Their flexibility allows for the incorporation of covariates, such as age or renal function, to explain variability, enhancing precision in dose selection and the optimization of therapeutic regimens [13].

It is worth mentioning that there exist more basic pharmacometric tools and approaches, often serving as starting points for more complex analyses or constituting a part of more complex models. Included in this group are allometric scaling, which helps in extrapolating the PK and PD characteristics of a drug across different species, and sensitivity analysis, which aids in parametrizing PD models. NCA, for instance, is used as a starting point in selecting initial parameter values for PK analyses, as well as in bioequivalence studies. This section does not provide details on specific pharmacometric methods and their mathematical foundations, as this is not the objective of the current review paper. For a deeper understanding of modeling techniques and their application, we recommend other review papers that explain this topic from the basics [7,8,9,10,66].

Pharmacometrics is essential at all stages of drug development, from selecting a drug target to clinical trials, as well as in the optimization of existing treatments. Frequently, pharmacometric tools are used in combinations that enhance their impact and allow for a more complex and detailed description of a drug’s PK, PD, and influence on DisP [64,68,69]. As presented in this article, by incorporating individual patient characteristics, disease variability, and complex drug–disease interactions, these methods offer a pathway towards the development of novel treatment modalities and strategies, as well as more effective, safe, and personalized therapies for AIDs.

## 5. Pharmacometrics in Pre-Clinical Studies on Autoimmune Diseases

Pharmacometric methodologies are increasingly utilized as useful tools to analyze and interpret pre-clinical in vitro and in vivo data at the early stages of drug development for AIDs. PBPK, PK/PD, PK, DisP, QSP, and population models represent essential mathematical tools for predicting drug behavior in the animal body and assessing both efficacy and safety, all prior to human trials.

A series of studies by Earp and coworkers, published in 2008 and 2009, provides a comprehensive quantitative assessment of arthritis progression, dexamethasone (DEX)’s PK and PD, interplay among inflammatory mediators, corticosterone concentrations, bone mineral density, and paw edema in rat models of RA. DEX is one of the synthetic corticosteroids commonly used in the treatment of RA, and corticosterone is an endogenous corticosteroid produced by the adrenal glands of rats. In the first study, the researchers aimed to assess whether inflammation in a rat model of RA alters the PK of DEX compared to healthy controls [70]. The study employed NCA and popPK modeling in NONMEM VI software (NONMEM Project Group, University of California, San Francisco, CA, USA). The study concluded that although there was a statistically significant difference in clearance between healthy and arthritic rats, the difference was minor and unlikely to affect DEX disposition substantially in arthritic rats. In another publication, the same research group aimed to identify a rat model of arthritis with the least inter-animal variability to improve study designs for arthritis research [71]. To this end, NLME modeling was utilized. The model included components for the production, loss, and feedback of edema. The study focused on collagen-induced arthritis (CIA) and adjuvant-induced arthritis (AIA) animal models of RA in Lewis and Dark Agouti rats. The results indicated that Dark Agouti rats may provide a more dynamic range of edema response than Lewis rats, and the onset time of the disease varied significantly between groups, which should be considered in future studies.

Two subsequent studies aimed to develop mechanism-based PK/PD and DisP models to describe the progression of arthritis in rats, focusing on the time-course of various biomarkers and disease endpoints, such as expression of glucocorticoid receptors, TNFα, IL-6, and IL-1β, as well as paw swelling and bone mineral density. The model was developed by first fitting molecular biomarkers (cytokine mRNA, glucocorticoid receptor mRNA, and plasma corticosterone) and fitting disease outcomes (paw edema and bone mineral density). The model included equations for glucocorticoid receptor mRNA turnover and bone mineral density based on different bone types. It effectively characterized the delay in cytokine mRNA responses and the subsequent effects on paw swelling and bone mineral density, demonstrating the complex interrelations among various signaling molecules and disease progression [44]. The next study aimed to develop a mechanistic PK/PD/DisP model to understand the effects of DEX on DisP in a rat model of RA, focusing on factors responsible for edema and bone loss. The study employed indirect-response models, drug interaction models, transduction processes, and the previously developed fifth-generation model of corticosteroid dynamics [72]. The analyses were performed in S-ADAPT software (BMSR, University of Southern California, Los Angeles, CA, USA). The model integrated pro-inflammatory cytokine mRNA, glucocorticoid receptor mRNA, plasma corticosterone, paw edema, and bone mineral density. It assumed that DEX binds to the same receptor as endogenous corticosterone, mediating the observed responses. It was demonstrated that lower doses of DEX can effectively suppress key cytokines related to bone erosion, suggesting that optimal dosing can mitigate adverse effects on bone mineral density while controlling inflammation [73].

In 2011, a study by Lon and colleagues aimed to develop a PK/PD/DisP model to assess the impact of etanercept, a biologic drug that acts as a TNFα inhibitor, on RA progression in CIA [64]. CIA rats were administered etanercept either IV or subcutaneously (SC), and its plasma concentration– and effect–time profiles were quantified. Pharmacological effect was observed as changes in paw swelling. The data obtained were then applied to successfully model the PK, PD, and DisP using ADAPT 5 software (BMSR, University of Southern California, Los Angeles, CA, USA). The study concluded that etanercept modestly reduces paw swelling in CIA rats, with maximum inhibition of paw edema of 28.9%, and a half-maximal inhibitory concentration (IC_50_) of 22.7 µg/mL. The PK/PD/DisP model effectively described the drug’s effects, suggesting its potential applicability to other anti-cytokine biologic agents for RA.

Liu and coworkers presented a popPK/PD/DisP model to evaluate the effects of anakinra, another biopharmaceutical medication that acts as a recombinant human IL-1 receptor antagonist [69]. The study involved administering anakinra to rats through SC infusion at varying doses and durations. The swelling of the hind paws was monitored as an indicator of DisP. The PK/PD parameters for the different study groups were estimated using the NLME modeling software NONMEM (Icon, USA). The PK profiles of anakinra were described using a two-compartment model with two sequential absorption processes and linear elimination. The DisP and drug effects were modeled using a transduction-based feedback model with a logistic growth rate and an indirect-response model. The model effectively captured the PK and paw swelling data, showing that anakinra had modest effects on paw edema in CIA rats, with maximum inhibition of 28% and an IC_50_ of 49.4 ng/mL.

A popPK/PD/DisP approach was used by Song and Jusko to assess sex differences in the PK/PD of DEX using a CIA model in rats [63]. The research involved comparing paw size in male and female rats across four groups: healthy controls, non-treated arthritic animals, and arthritic animals treated with DEX at two different dose levels. The study applied a DisP model in combination with a minimal PBPK model for drug disposition, and an inhibitory indirect-response model, all within a population modeling framework. The results revealed that DEX clearance was 43% higher in male rats, although other PK parameters were similar between sexes. Female rats exhibited faster DisP, peak edema, and remission. DEX effectively suppressed paw edema in both sexes with equal capacity; however, it was less potent in females, as indicated by higher IC_50_ values. The study provided a comprehensive evaluation of sex differences in DEX’s PK and PD in CIA rats, offering insights for more detailed assessments of sex, drug, and disease interactions in RA.

PK/PD/DisP modeling may also be successfully used to assess potentially beneficial PD interactions between anti-inflammatory drugs. In a study by Xiaonan Li and colleagues, immunosuppressive and anti-inflammatory effects of DEX given in combination with a non-steroidal anti-inflammatory drug, naproxen, were assessed [74]. A comprehensive PK/PD/DisP model was used to describe the PK and time-course of single and combined anti-inflammatory effects (changes in paw edema) of dual-drug inhibition, while also considering sex differences in CIA rats. The model revealed additive effects when combining DEX and naproxen. Simulations performed using this model highlighted the potential of naproxen to reduce the dose of steroids in CIA rats. However, the combination therapy showed more pronounced beneficial effects in males compared to females. Several similar PK/PD studies were performed to evaluate the efficacy of various therapeutics, including small molecules as well as biologics, for the treatment of RA [62,75,76,77].

In 2022, Świerczek and coworkers published an article describing the effects of a novel dual phosphodiesterase (PDE)4/7 inhibitor in an encephalomyelitis model of MS in mice [65]. They presented a simple DisP model that captured the initial sudden onset of the disease after 7 days following the immunization of mice with a myelin oligodendrocyte glycoprotein (MOG) [35,36,37,38,39,40,41,42,43,44,45,46,47,48,49,50,51,52,53,54,55], partial remission of the disease, and its subsequent relapse. They assessed the potency (IC_50_) of the investigated compound in the inhibition of DisP, which was quantified using a clinical score value. A PK/PD modeling approach was also used by this research group in a publication assessing the effects of PDE inhibitors in the CIA model of RA in rats [78]. By using a mouse model of AIH induced by IV administration of concanavalin A and a mechanistic PK/PD modeling approach, selective PDE3, PDE4, and PDE7 inhibitors, as well as non-selective PDE inhibitors, were evaluated as potential medications for AIH [41,79]. In these studies, various biomarkers of inflammation and liver damage were utilized, such as serum IL-6, TNFα, IL-10, and IFN-γ, as well as the activities of alanine transaminase (ALT) and asparagine transaminase (AST). This approach enabled the development of mechanistic PK/PD models to assess the impact of inhibition of individual PDE types on the development and progression of AIH in mice.

A work by Haselmayer et al. presented research on M2951, a new Burton’s tyrosine kinase (BTK) inhibitor, indicating its potential to treat AIDs such as RA and SLE by inhibiting immune cell activation [80]. They used various in vitro and in vivo methods to test M2951, including cellular assays and disease models in mice, to demonstrate its efficacy and build a PK/PD model linking BTK inhibition to disease severity reduction. The study used PK/PD modeling to show that specific levels of BTK occupancy (60% and 80%) were associated with significant and near-complete disease inhibition, respectively, in RA and SLE models. The summarized information on the example pre-clinical studies described in this section is provided in Table 3.

In summary, pharmacometrics plays an important role in pre-clinical drug discovery and development by providing advanced methodologies to analyze and interpret in vivo and in vitro data, thereby predicting drug behavior, efficacy, and safety before human trials. The various modeling approaches facilitate the assessment of PK, PD, and DisP, as demonstrated in studies evaluating various therapeutics for autoimmune conditions. By enabling the prediction and assessment of drug effects, sex differences in PK and PD, and drug interactions, pharmacometrics offers a quantitative and more informed, precise, and efficient approach to drug evaluation, discovery, and development compared to traditional statistical methods.

## 6. Translational Pharmacometric Approaches in Autoimmune Diseases

Pharmacometrics plays an increasing role in translating results from pre-clinical investigations into clinical applications. In AIDs, where individual patient responses can vary greatly, pharmacometrics is expected to aid in predicting how both small molecules and biologics might behave in humans based on the results of pre-clinical studies. This section describes the role of pharmacometrics in translating the results of pre-clinical animal studies to potential clinical applications. This involves examining drug E-R relationships and integrating data from both in vivo and in vitro models. It also highlights the challenges in ensuring the translatability of these models and the necessity of considering various factors, such as disease stages, animal models, medications’ mechanisms of action, and patient-specific responses.

A study by Wong and colleagues focused on the effectiveness of rodent immune-mediated arthritis models in predicting the therapeutic activity of anti-arthritis agents [81]. It highlighted the high attrition rates in drug discovery, especially in Phase II clinical trials, due to the lack of efficacy of potential treatments. This emphasizes the need to improve the predictability of pre-clinical disease models not only for RA but also for other immune-related disorders and AIDs. The use of rodent models, particularly in studying the mechanisms of inflammatory joint disease, was discussed as a valuable approach. In terms of methods, the study used male Lewis rats for the AIA and CIA animal models of RA. The efficacy of various treatments was assessed using ankle diameter measurements. In addition, PK analyses of the studied medications were performed. The results section described administering a range of doses of several drugs, including indomethacin, methotrexate, etanercept, tofacitinib, and DEX, to rats with established AIA and CIA. The detailed outcomes for each drug were outlined, providing insights into the dose–efficacy relationship in these pre-clinical rat arthritis models. The practical application of this research lies in its potential to enhance the translation of pre-clinical findings to clinical settings, particularly for RA treatments, by better understanding the efficacy of various drugs in rodent models [81].

A study by Dowty et al. describes the translation of pre-clinical studies to clinical trials for tofacitinib, a Janus kinase inhibitor (JAK), in RA [82]. It focuses on determining dosing regimens that provide maximum therapeutic benefit with minimum toxicity using NCA analysis, direct Emax models, and PK simulations. The study used pre-clinical evaluations in a mouse CIA model of RA and clinical PK/PD profiles obtained from pooled data from four Phase II clinical trials in patients with RA. It was concluded that efficacy of tofacitinib in RA is driven by its IC_50_, however continuous daily inhibition is not required to maintain efficacy.

A study by Zheng et al. investigated MTRX1011A, a humanized anti-CD4 monoclonal antibody (mAb) with improved binding to the neonatal Fc receptor (FcRn) due to an N434H amino acid substitution [83]. This modification was expected to enhance the lifespan of antibodies by protecting them from lysosomal degradation. The study compared MTRX1011A with its predecessor, TRX1, in terms of PK and PD in both pre-clinical and clinical settings. The modeling aimed to quantitatively characterize the properties of MTRX1011A in comparison with TRX1. Although there was a large variability in the observed data, the model adequately described individual PK and PD profiles. The study found that the N434H mutation did not significantly alter the non-specific elimination rate or the PK/PD relationship of MTRX1011A compared to TRX1, and the authors concluded that while the PK/PD relationship of MTRX1011A in humans is similar to that of TRX1, the expected benefit from enhanced FcRn binding was not evident in the clinical setting. However, the presence of preexisting antibodies in RA patients recognizing the N434H mutation could have interfered with binding of MTRX1011A to FcRn, potentially impacting the results. The study highlighted the challenges in translating pre-clinical findings to clinical applications, especially due to variability in human populations and disease states. The results provided valuable insights into the development of engineered mAbs with improved PK profiles, emphasizing the need for careful evaluation in clinical studies [83].

A study by Biliouris and collogues assessed BIIB059, an antibody against the blood dendritic cell antigen 2, which had been developed to treat SLE [56]. The researchers used PK and PD data from cynomolgus monkeys to build a model predicting how the drug may behave in humans. They tested various doses of BIIB059 in monkeys and proposed a mechanistic PK/PD model that was subsequently scaled up by using allometric scaling of PK as well as sensitivity-analysis-driven scaling of the PD parameters to predict human outcomes. The predictions of the model matched the actual clinical results, suggesting that this method can help to select safe doses for first-in-human trials.

Rozanolixizumab is a fully humanized, high-affinity anti-human FcRn mAb designed to inhibit IgG recycling and reduce circulating IgG levels [84]. The purpose of a study by Lledo-Garcia and colleagues was to develop a PK/PD model to predict human responses to rozanolixizumab based on pre-clinical data, and to refine this model using first-in-human data [85]. The study used a popPK/PD modeling approach to determine the relationship between IgG response and rozanolixizumab concentration over time. The proposed structural model was a mechanistic PK/PD model that described the PK of rozanolixizumab using a two-compartment model with TMDD and the PD by linking drug concentration to IgG reduction. The proposed model accurately predicted human responses to rozanolixizumab, especially at 4 and 7 mg/kg doses, and was refined using first-in-human data to improve its predictive performance. This model may be used to inform future clinical trial designs, optimize dosing regimens, and examine hypotheses related to disease management and treatment, with ongoing updates as more clinical data become available.

mRNA-6231 was designed to express the HSA-IL2m protein, which at low concentrations and a prolonged half-life selectively stimulates the expansion of regulatory T cells (Tregs) [86]. The purpose of the article by Rajlic and coworkers was to develop a mechanistic kinetic–pharmacodynamic (KPD) model to describe the temporal patterns and dose-dependent changes in HSA-IL2m protein levels and Treg expansion following single and repeated administrations of mRNA-6231 in non-human primates, and to scale this model up to predict human responses. The proposed KPD model used ODEs to describe the distribution and effects of mRNA-6231. It incorporated a virtual transit compartment to account for delays in dynamic systems and used an Emax model to relate mRNA concentration to HSA-IL2m synthesis. Significant covariates included body weight, which influenced the absorption rate constant, clearance of HSA-IL2m, and clearance from the transit compartment. The primary PD markers used were the plasma concentrations of HSA-IL2m and the percentage of Treg cells. The study concluded that the mechanistic KPD model could reliably predict the PD response to mRNA therapeutics in humans, aiding in the selection of appropriate doses for clinical trials [86].

The studies presented in this section, employing pharmacometric approaches in the translation of pre-clinical studies into clinical applications, are summarized in Table 4.

As shown in this section, pharmacometrics is pivotal in translational science, especially in bridging the gap between pre-clinical findings and clinical applications. By employing mathematical models, pharmacometrics allows for the extrapolation of drug behavior observed in animal models to predict human responses, optimize dosing regimens, and improve the design of clinical trials. This approach is particularly valuable in AIDs, where patient responses can vary significantly and a high attrition rate of drug candidates in Phase II clinical trials is observed due to lack of efficacy. By understanding drug interactions with the complex immune system and tailoring therapies to individual patient needs, pharmacometrics enhances the predictability of therapeutic efficacy and safety, thereby facilitating a more efficient and targeted transition from laboratory bench to bedside [87].

## 7. Population Modeling and Simulation in Clinical Applications for Autoimmune Diseases

Population models are frequently used in the evaluation and optimization of therapies for cancer drugs and antimicrobial treatments, both by research groups and clinicians [88,89]. These models analyze drug concentration–time, response–time and, in some cases, E-R data from diverse patient populations to understand variability in drug disposition and response. They enable personalized medicine by identifying covariates, which explain variability in drugs’ PK and PD. This section presents examples of the application of population modeling in advancing therapies for AIDs in the clinic.

### 7.1. Rheumatoid Arthritis

#### 7.1.1. Small Molecules for Rheumatoid Arthritis

Small-molecule DMARDs have a long-standing role in the management of RA, offering disease-modifying effects that slow progression and improve patient outcomes, forming the backbone of RA treatment strategies over decades [90]. A study by Wojciechowski and coworkers aimed to describe how disease activity in RA changes in response to triple DMARD therapy including methotrexate, sulfasalazine, and hydroxychloroquine (HCQ), and to develop a model that can predict individual patient outcomes, potentially leading to personalized treatment strategies [91]. The study used population-based DisP modeling to describe changes in DAS28 over time, employing various structural DisP models like linear, quadratic, and exponential functions to fit the data. Simulations were performed to evaluate the performance of the models and to understand the impact of residual unexplained variability on DAS28 measurements. An exponential model was proposed as the best fit for describing the decline in DAS28 over time, with a typical population half-life of 6.2 weeks for non-smokers and 10.4 weeks for smokers. Significant covariates included age, smoking status, and corticosteroid therapy, which influenced the variability in DAS28 time-course and the rate of response to treatment. The study concluded that population modeling could help to identify patients with poor disease trajectories early, potentially leading to more effective personalized treatment strategies [91].

Model-based approaches have advanced the optimization of small-molecule kinase inhibitors for RA, supporting precise dosing and improved therapeutic efficacy [92,93,94]. A study by Toyoshima and colleagues developed models to predict the efficacy of peficitinib, a Janus kinase (JAK) inhibitor used for the treatment of RA, by examining its impact on ACR20 response rate and DAS28-CRP measurements in patients with RA [95]. The researchers used NLME models with NONMEM software to analyze the data, evaluating various covariates through stepwise forward addition and backward elimination methods [66]. Two models were proposed: a continuous-time Markov model for ACR20 response rates, and an indirect-response model for DAS28-CRP measurements. The models effectively described the treatment response over time and highlighted that baseline disease severity was correlated with the magnitude of the treatment response, suggesting no need for dose adjustment.

A study by Chan et al. aimed to use a Model-Informed Drug Development (MIDD) approach to analyze the clinical efficacy data of fenebrutinib, a BTK inhibitor, in patients with RA [96]. The study used popPK modeling, E-R analysis, and MBMA to understand the drug’s effects and optimize its development. A three-compartment model with linear elimination and a flexible absorption transit compartment model were proposed to describe the PK of fenebrutinib. The study used ACR20, ACR50, and ACR70 improvement criteria, as well as DAS28, as clinical outcomes to evaluate treatment responses. The MIDD approach enabled a robust interpretation of Phase II clinical trial data, showing that fenebrutinib achieved an efficacy plateau within the exposure range tested, and its effects were consistent with historical data for similar treatments. This model can be used to guide dose selection and regimen optimization in future clinical trials.

Upadacitinib is a selective JAK1 inhibitor developed for treating moderate-to-severe RA and other AIDs. The purpose of the article of Klünder et al. was to characterize the popPK of upadacitinib across Phase I–III clinical trials using data from both immediate-release (IR) and extended-release (ER) formulations [97]. A two-compartment model was proposed, which included first-order absorption with lag time for the IR formulation, mixed zero- and first-order absorption with lag time for the ER formulation, and linear elimination. Significant covariates included population (RA subjects vs. healthy subjects), creatinine clearance and baseline body weight on clearance, and body weight on volume of the central compartment. The developed popPK model could adequately describe upadacitinib’s plasma concentration–time profiles, with no clinically meaningful effect from the identified covariates on upadacitinib exposures. This model may be used for simulations and to evaluate the E-R relationship of upadacitinib, aiding in optimizing dosing regimens for different patient populations.

#### 7.1.2. Biologic Drugs for Rheumatoid Arthritis

In recent years, biologic drugs have played an increasingly important role in the treatment of RA, as well as other AIDs, by specifically targeting immune pathways involved in inflammation, improving disease management, and offering treatment options for patients who do not respond adequately to traditional therapies [98].

Olokizumab is an mAb directly inhibiting IL-6 [99]. The article of Kretsos et al. aimed to explore the PK and PD of olokizumab in patients with mild-to-moderate RA to identify suitable dosage regimens for subsequent Phase II studies [100]. The study employed model-based adaptive design, including reverse engineering of first-in-class PK/PD models, Bayesian analysis, and the application of a multidimensional desirability index for optimal study design. Data were obtained from a first-in-patient, randomized, double-blind, placebo-controlled study on patients with mild-to-moderate RA. The model successfully defined a dose window for CRP suppression and confirmed the attainment of the study objectives through interim and final analyses.

A study by Schmidt and colleagues aimed to develop a Mechanistic Axes Population Ensemble Linkage (MAPEL) algorithm to calibrate virtual patient populations whose simulated responses matched clinical trial outcomes, and to explore alternate hypotheses reflecting population biology uncertainty [101]. The data analyzed included simulated ACRN index responses from virtual patient cohorts, calibrated to match clinical trial data for approved therapies. The source of the data was clinical trials focusing on patients who do not respond well to methotrexate. The MAPEL algorithm identified IFNβ as a key mechanistic contributor to disease state variations in response to rituximab. The study concluded that the MAPEL algorithm can propose mechanistic hypotheses for differences in clinical populations and facilitate the development of multi-analyte biomarkers prognostic of patient responses in silico.

An article by Chakraborty and coworkers aimed to analyze the PK and PD properties of canakinumab, a human anti-interleukin-1β mAb, focusing on its clinical development for treating cryopyrin-associated periodic syndromes (CAPSs) and other immune-mediated diseases, such as RA, asthma, and psoriasis [102]. Canakinumab showed PK properties typical of an IgG1 mAb, with slow serum clearance and a long elimination half-life. It displayed linear PK with a dose-proportional increase in exposure. The study concluded that canakinumab has high SC bioavailability and found no evidence of accelerated clearance or time-dependent changes in PK after repeated administration.

Golimumab is a human immunoglobulin G1k (IgG1k) mAb that binds with high affinity to both transmembrane and soluble forms of TNFα [103]. A study by Hu et al. aimed to develop a mechanistically interpretable approach to population E-R modeling to characterize the PK/PD of golimumab in patients with RA, using changes in ACRN as a clinical outcome [104]. A sequential popPK/PD modeling approach was used by first fitting the serum golimumab concentration–time data to the PK model, and then fitting the ACRN–time data to the PD model, implemented in the NONMEM software. An indirect-response PK/PD structural model was proposed, which viewed TNFα as the precursor of the cascade of secondary mediators involved in RA’s development. The final population model incorporated covariates such as body weight, age, sex, race, disease duration, and use of concomitant medications, describing their impact on the PK and PD parameters of golimumab. The proposed approach was expected to enable wider applications of indirect-response models in clinical trials, especially when a wide range of dose levels is investigated.

Canakinumab, an IL-1β inhibitor, reduces inflammation by blocking IL-1β signaling in RA patients [105]. A study by Ait-Oudhia et al. aimed to develop popPK/PD models to describe the responses to canakinumab in patients with RA, and to explore the potential benefits of different dosing regimens of this medication [42]. The study used a two-compartment model for canakinumab PK and a quasi-equilibrium model for its binding to IL-1β, incorporating simulations to predict clinical outcomes and dose–response relationships. The final PK/PD model linked total canakinumab and IL-1β concentrations to CRP plasma concentrations and ACR scores, using free IL-1β as the PD driver. Body weight was identified as a significant covariate affecting the clearance and volume of distribution for canakinumab and IL-1β. The study concluded that canakinumab at 150 mg every 4 weeks improves RA symptoms, but higher doses do not provide additional benefits, and the model can be adapted for other inflammatory diseases and AIDs.

Tocilizumab is a humanized mAb that targets the IL-6 receptor [106]. The study of Frey and coworkers aimed to develop a popPK model for this medication to describe its PK variability among individuals, and to assess the influence of different factors on PK parameters [107]. A two-compartment structural model with parallel linear and non-linear (Michaelis–Menten) elimination kinetics was proposed to describe the serum concentration–time profile of tocilizumab in patients with RA. Significant covariates affecting the PK parameters included body surface area, sex, high-density lipoprotein cholesterol, and the logarithm of RF on clearance; total protein and albumin on the central volume of distribution; and albumin, creatinine clearance, and smoking on the maximum elimination rate. The final PK model accurately described the PK characteristics of tocilizumab and identified key covariates influencing its PK, providing valuable insights for optimizing dosing regimens in patients with RA.

A study by Levi and coworkers investigated the relationship between tocilizumab exposure and its efficacy in treating RA by analyzing data from four Phase III clinical trials [108]. Graphical analyses were used to assess the relationships between tocilizumab and key clinical endpoints, and a popPK/PD model was developed to explore the relationship between tocilizumab exposure and efficacy, quantified by the DAS28. The proposed model was an indirect-response model with sigmoid inhibition of DAS28 production by tocilizumab, estimating parameters including baseline DAS28, first-order rate loss of DAS28 score, and the maximum inhibitory effect of tocilizumab. Significant covariates included IL-6 concentrations, sex, race, HAQ score, Global Pain Score, and physician’s global score of disease activity. The study concluded that tocilizumab effectively reduced DAS28 scores, with a maximum reduction of 5.0 units in males and 4.6 units in females, and that the presence of neutralizing anti-tocilizumab antibodies did not affect the PK/PD model’s outcomes.

The purpose of an article written by Bastida and coworkers was to investigate the relationship between tocilizumab serum concentrations and response in RA patients, focusing on the dynamics of individual components of composite disease activity measures [43]. The study used NLME models implemented in NONMEM software to evaluate the relationship between drug exposure and response, employing a previously published PK model and an indirect-response model for DAS28, SDAI, and CDAI. A direct Emax model was proposed to describe the relationship between tocilizumab serum concentrations and disease activity measures. The PD markers used in the study included CRP, ESR, tender joint count, swollen joint count, patient global assessment, and evaluator global assessment. The study’s results revealed that patients achieving DAS28 remission with tocilizumab might still have significant residual clinical disease activity due to the high weight of inflammatory markers in the DAS28 formula, unlike SDAI and CDAI. The modeling approach confirmed the need for higher serum drug concentrations to normalize clinical variables compared to inflammatory markers.

Sarilumab is a medication used to treat RA that binds to the IL-6 receptor with high affinity, blocking both *cis* and *trans* signaling pathways of IL-6 [109]. A study by Xu aimed to develop a popPK model for sarilumab and to identify patient characteristics that affect the PK of the drug [110]. The researchers used a two-compartment model with first-order absorption and both linear and non-linear Michaelis–Menten elimination to describe sarilumab’s PK. The study’s results indicated that body weight has limited clinical relevance to sarilumab exposure, and that no dose adjustment is required based on body weight or other demographics. The model accurately described the PK of sarilumab in patients with RA.

An article by Ma and coworkers presented popPK/PD models to describe the effects of sarilumab on DAS28-CRP and absolute neutrophil count (ANC) in patients with RA [111]. The researchers used mechanistic models to simulate how sarilumab impacts DAS28 and ANC over time, validating these models with real patient data. The proposed model linked sarilumab concentrations to disease activity and ANC, using parameters describing drug effects and elimination rates to predict outcomes. They concluded that 200 mg of sarilumab every 2 weeks was more effective than 150 mg, and that no dose adjustments were needed based on patient characteristics, supporting the higher dose as the starting point.

Adalimumab (Humira) and its biosimilars are TNFα inhibitors used in the treatment of RA that act by reducing inflammation and prevent joint damage [112]. The study of Kang et al. aimed to assess the PK similarity between adalimumab-adbm and Humira in patients with RA, and to evaluate the impact of switching from Humira to adalimumab-adbm on adalimumab’s PK [113]. The study utilized popPK models developed through NLME modeling to analyze adalimumab concentration–time data from different treatment arms, including adalimumab-adbm and Humira. A two-compartment model with sequential zero- and first-order absorption and linear elimination was proposed to describe the PK of adalimumab in both healthy subjects and RA patients. Significant covariates affecting adalimumab’s clearance included body weight, ADAs, baseline RF, CRP concentrations, and albumin levels. The study concluded that adalimumab-adbm is pharmacokinetically similar to Humira in RA patients, and that switching from Humira to adalimumab-adbm does not impact adalimumab’s PK.

The study of Cheryl Li et al. aimed to describe the PK/PD relationship of PF-04236921, an anti-IL-6 mAb, in healthy volunteers and patients with RA, SLE, and CD [114]. NLME modeling was utilized, which included data from five clinical studies to develop integrated popPK and popPK/PD models. The models were refined by incorporating covariates and using techniques such as bootstrap runs and prediction-corrected visual predictive checks (pcVPCs) to validate the final parameter estimates. A two-compartment model with first-order absorption and linear elimination was proposed for the PK, while an indirect-response model was used to describe the PK/PD relationships. It was found that the clearance of PF-04236921 was higher in CD patients compared to other populations, and factors such as baseline albumin and CRP levels significantly impacted the efficacy of the drug. The study concluded that the proposed integrated popPK and popPK/PD models enable the simulation of the PK and PD profiles of PF-04236921 under various dosing regimens and patient populations, aiding in future clinical studies of anti-IL-6 mAbs [114].

Abatacept is a selective T-cell co-stimulation modulator used in the treatment of RA to inhibit T-cell activation [115]. The study of Li et al. aimed to develop popPK and exposure–response models for abatacept in patients with RA, using data from Phase II and III clinical trials [116]. The researchers used a two-compartment model with first-order elimination to describe the PK of abatacept, and they performed simulations to predict drug concentrations and responses. They also used an ordered categorical proportional-odds model to assess the probability of achieving different levels of clinical response (ACR20, ACR50, ACR70) based on drug exposure. The proposed popPD model was an Imax model, which described the relationship between abatacept exposure and the reduction in DAS28. Baseline body weight was the only clinically relevant covariate affecting abatacept’s clearance and volume of distribution. The study concluded that abatacept’s efficacy increases with higher steady-state trough concentrations, with a near-maximal response at 10 mg/mL. The E-R relationship was consistent for both IV and SC administration routes. The proposed model can be used to optimize abatacept dosing regimens, ensuring that patients achieve the target therapeutic exposure for maximum efficacy [116].

A study by Nakada and Mager aimed to characterize cytokine and CRP profiles using a mathematical model to predict therapeutic resistance to biologics in RA patients [117]. The study analyzed CRP concentrations and cytokine baselines using data from the RISING study and other clinical datasets for validation. The model’s predictions implied that high baseline IL-1β and targeted cytokines could predict therapeutic resistance. It also showed robust performance across various datasets, offering a platform to design novel treatments for RA.

### 7.2. Systemic Lupus Erythematosus

#### 7.2.1. Small Molecules for Systemic Lupus Erythematosus

Mycophenolic acid (MPA), an active form of mycophenolate mofetil (MPM), is a potent, selective, non-competitive, and reversible inhibitor of inosine monophosphate dehydrogenase. It blocks the de novo pathway of guanosine nucleotide synthesis without incorporating itself into DNA [118]. Since the de novo purine synthesis pathway is crucial for the proliferation of T and B lymphocytes, while other cell types can utilize alternative synthesis pathways, MPA exerts a cytostatic effect more strongly on lymphocytes than on other cell types. In a study by Sherwin et al., a popPK model was developed for MPM [119]. The proposed model incorporated enterohepatic recycling to aid in individualized dosing for pediatric and adolescent SLE patients. Body weight was found to influence the apparent oral clearance and the apparent distribution volume of MPM, as well as the apparent clearance of its main inactive metabolite 7-O-MPA-β-glucuronide, when allometric scaling was applied.

A study by Yang and colleagues aimed to outline various pharmacometric approaches related to PK that are used to optimize the existing treatment of SLE to improve clinical outcomes with various small-molecule therapies, including immunosuppressants and immunomodulators, such as methotrexate, azathioprine, cyclophosphamide, mizoribine, fludarabine, MPA, cyclosporin A, and IV immunoglobulin [120]. The study proposed using popPK and PBPK models to describe drug behavior and variability in patients, and to facilitate real-time dose adjustments using Bayesian estimators. For methotrexate, significant covariates included patient demographics and disease status, which influenced drug absorption and clearance. For azathioprine, genetic factors, such as polymorphisms in drug-metabolizing enzymes, were significant covariates affecting drug metabolism and distribution parameters. MPM’s PK was influenced by renal function and co-medications, which impacted drug absorption and clearance parameters. Cyclophosphamide’s PK parameters were affected by liver function and patient age, which influenced drug metabolism and distribution [120].

The study of Yao et al. aimed to speed up the early clinical development of a new drug, teriflunomide sodium, for treating SLE by using data from leflunomide [121]. A popPK model was developed using PK data from both healthy volunteers and patients with RA. The model was validated using various diagnostic plots, VPC, and bootstrap methods to ensure its accuracy. A one-compartment model with enterohepatic circulation characteristics was proposed to describe the PK of teriflunomide after administration of leflunomide and teriflunomide sodium. The developed PopPK model effectively described the PK of teriflunomide in both healthy subjects and patients, and it can be used to support the design of Phase II clinical trials for SLE. The study found that body weight significantly affected the apparent volume of the central compartment. Male sex was found to increase the apparent volume of the central compartment by 21% compared to females. The genetic polymorphism significantly affected both the absorption rate and systemic clearance of teriflunomide. Heterozygotes (ABCG2 34GA) and mutant homozygotes (ABCG2 34AA) showed increased absorption rates (by 51% and 97%, respectively) and increased clearance (by 37% and 111%, respectively) compared to the wild-type individuals (ABCG2 34GG) [121].

The study of Scheetz et al. aimed to assess the impact of HCQ shortages on patients with SLE, and to model different dosing strategies to manage these shortages during the COVID-19 pandemic [122]. The researchers used Pmetrics for R to simulate HCQ concentrations every 2 h until concentrations dropped below 30 ng/mL, employing Monte Carlo simulations to assess variability and predict outcomes under different dosing scenarios. The model simulated three HCQ dosing strategies: continuing the full dose until depletion, alternating full and half doses, and taking half doses daily to extend the supply. This approach aimed to predict how long blood HCQ levels would stay above a therapeutic threshold. Significant covariates included baseline blood HCQ concentration, which influenced how long patients could maintain therapeutic levels under different dosing strategies. The study’s results indicated that rationing HCQ by taking half doses could extend the duration of therapeutic levels, but the effectiveness of this strategy depended on the patient’s baseline HCQ concentration. This model can help clinicians make informed decisions on HCQ dosing strategies during drug shortages, potentially reducing the risk of disease flares in SLE patients [122].

#### 7.2.2. Biologic Drugs for Systemic Lupus Erythematosus

Anifrolumab is an anti–interferon-α receptor subunit 1 (anti-IFNAR1) mAb. Chia and coworkers published a study on anifrolumab, focusing on its optimal dosage, safety, and efficacy [123]. The researchers used a population approach to analyze the E-R relationship, PK, and SRI(4) efficacy data. A popPK model was developed to describe drug disposition and to identify covariates affecting its clearance. The study found that patients with higher levels of type I IFNGS and those with a higher body weight had a significantly higher clearance of anifrolumab. The study concluded that understanding the E-R relationship of anifrolumab may aid in selecting the optimal dosage regimen for Phase III studies in SLE patients.

A study by Almquist et al. aimed to evaluate how various covariates impact the PK of anifrolumab, and to inform its use in clinical practice [124]. The researchers developed a popPK model to analyze data from five clinical trials, employing a stepwise covariate model-building process to identify significant covariates [66]. A two-compartment model with parallel first-order elimination pathways and time-varying clearance was proposed to describe the PK of anifrolumab. The model was validated using data from multiple studies. Significant covariates included body weight and baseline IFNAR1 level. The study concluded that individualized dosing approaches guided by PK algorithms could be safer, more efficient, and cost-effective for treating SLE patients.

Belimumab is an mAb targeting the B-lymphocyte stimulator (BLyS). The study of Dimelow and coworkers aimed to understand and describe how belimumab increases circulating memory B-cell levels in patients with SLE, and to explore the effects of dose, demographics, and disease characteristics on this effect [125]. The study used a combination of Bayesian and maximum likelihood methods to develop and refine models predicting memory B-cell dynamics. The final model included baseline BLyS and anti-dsDNA as significant covariates and was used to simulate memory B-cell responses under different dosing regimens of belimumab. It was postulated that belimumab likely increases circulating memory B-cell levels by stimulating their trafficking from lymphoid and inflamed tissues into the blood, with baseline BLyS and anti-dsDNA levels having a minimal impact on predicting the response size and duration [125].

Dapirolizumab pegol is a drug that blocks the interaction between CD40 and the CD40 ligand, which is important for immune responses in SLE [126]. The study of Acharya and coworkers aimed to understand the dose–exposure–response relationship of this medication [127]. A two-compartment model with first-order elimination was selected as the structural starting point for the popPK analysis. The only significant covariate in the popPK model was body weight, which influenced both clearance and volume of distribution. The E-R model assessed how different levels of dapirolizumab pegol in the blood affected the likelihood of patients with SLE transitioning between “non-responder” and “responder” status based on BICLA responder rates. Dapirolizumab pegol increased the probability of transitioning from “non-responder” to “responder” status, showing a positive exposure-dependent effect, and reduced the probability of transitioning back to non-responder status [127].

Atacicept is a drug that works by blocking two proteins: BLyS factor and a proliferation-inducing ligand, which are important for the survival, growth, and activity of B cells in the immune system [128]. A study by Pitsiu and coworkers developed a popPK model to describe how atacicept behaves in the body, using data from healthy volunteers and patients with SLE [129]. Body weight and baseline BLyS factor concentration were identified as significant covariates, but their effects on atacicept exposure were not clinically relevant. The model accurately described atacicept concentrations and variability, supporting the selection of suitable doses for further clinical development. The study confirmed that atacicept is well tolerated and effective in reducing SLE disease activity.

Anifrolumab is used in patients with moderate-to-severe SLE. The purpose of the article of Chia et al. was to describe the PK/PD relationship in patients treated with anifrolumab [130]. The proposed model was an indirect-response model that described how anifrolumab inhibited the production of type I IFN-inducible genes. The model was implemented using the NONMEM software. Significant covariates included body weight, which was inversely associated with anifrolumab concentrations, and high IFNGS expression, which was associated with lower systemic drug exposure. The PD marker used was the 21-IFNGS gene signature, which measures the expression of genes induced by type I interferon. Higher doses of anifrolumab (300 mg) resulted in rapid, substantial, and sustained neutralization of the 21-IFNGS, while lower doses (150 mg) produced delayed and variable effects. The results of the study indicated that higher drug exposure leads to better PD responses. This model can be used in future research to optimize dosing regimens for anifrolumab in different populations, such as pediatric patients, and to explore new routes of administration [130].

A study by Gao and coworkers aimed to understand the role and mechanism of conventional T cells (Tcons) and Treg cells in the inflammatory response of SLE, and to explore the potential of using the Tcon/Treg ratio to characterize SLE disease progression [131]. The study employed a mathematical model using a modified Lotka–Volterra equation. The study analyzed clinical data from previous studies on low-dose IL-2 therapy in SLE patients (NCT02465580, NCT02932137, NCT02084238, and DRKS00004858). The study found that low-dose IL-2 therapy can effectively reduce the Tcon/Treg ratio in SLE patients, suggesting a potential therapeutic strategy. The results indicated that IL-2 therapy could restore Treg functionality and reduce inflammation in SLE.

### 7.3. Multiple Sclerosis

Evobrutinib is a small-molecule BTK inhibitor used for the treatment of relapsing MS [132]. The purpose of the article of Papasouliotis and coworkers was to investigate the safety and efficacy of evobrutinib, explore its E-R relationships, and determine suitable dosing regimens for patients with relapsing MS [133]. The study used popPK/PD modeling to analyze data from patients treated with different doses of evobrutinib or placebo. The proposed PK model was a two-compartment model with sequential zero- and first-order absorption and first-order elimination, and an irreversible binding model for BTK occupancy. The main conclusions from the study were that evobrutinib exposure was significantly related to clinical outcomes, such as the reduction in T1 Gd and new/enlarging T2 lesions, and improvement in the annualized relapse rate. This model may be utilized to simulate alternative dosing regimens and optimize treatment strategies for patients with relapsing MS.

Daclizumab is a humanized IgG1 mAb that targets the alpha-subunit of the IL-2 receptor. The article of Diao and coworkers aimed to characterize the PK/PD relationships of daclizumab’s high-yield process in subjects with MS using data from four clinical trials [134]. NLME modeling was employed to analyze approximately 1400 subjects and 7000 PD measurements for each of three biomarkers. A sigmoidal Emax model was proposed to characterize CD25 occupancy, and an indirect-response model was used for CD56bright NK cell expansion. The Emax model for CD25 occupancy showed rapid saturation within 7 h, maintained at a serum concentration of 5 mg/L, and return to baseline in 24 weeks after the last dose. The indirect-response model for CD56bright NK cells showed expansion plateauing at week 36, with a maximum expansion ratio of 5.2, and returning to baseline within 24 weeks after the last dose. The PK/PD models were stable and performed well, indicating that daclizumab HYP effectively saturated CD25, expanded CD56bright NK cells, and reduced Tregs in MS patients. This model can be used to predict the effects of different dosing regimens of daclizumab HYP in MS patients.

Inebilizumab is a humanized mAb designed to target and bind to a specific protein called CD19, which is found on the surface of B cells. By binding to CD19, inebilizumab effectively depletes B cells from the bloodstream, which helps to reduce the concentration of autoantibodies [135]. The study of Yan et al. aimed to describe the PK of inebilizumab, an mAb used to treat AIDs such as neuromyelitis optica spectrum disorders (NOSDs), SS, and relapsing MS [136]. The researchers used a popPK model to analyze data from different studies involving inebilizumab. They employed NLME modeling to develop and validate the model. The proposed structural model was a two-compartment model with first-order elimination and a time-dependent non-linear elimination pathway. The PK of inebilizumab was accurately described by the proposed model. The clearance and distribution volume of inebilizumab were significantly influenced by body weight, indicating that heavier individuals had higher clearance and distribution volume of the drug. The presence of ADAs did not have a clinically relevant impact on the PK of inebilizumab. This model can be used to predict how inebilizumab behaves in different patient populations and individuals, helping to optimize dosing regimens and improve treatment outcomes for AIDs.

### 7.4. Special Populations

Pharmacometric studies in pregnant women and pediatric patients provide insights into PK variations, supporting dose adjustments tailored to these specific, vulnerable populations. A study by Balevic and coworkers aimed to understand how pregnancy affects the way in which HCQ is processed in the bodies of pregnant women with rheumatic diseases, including RA, and whether dose adjustments are needed during pregnancy [137]. The researchers used NLME modeling and performed simulations to compare drug concentrations during pregnancy and postpartum. The proposed model was a one-compartment model with fixed allometric scaling of weight on the volume of distribution and IIV of clearance. The study concluded that pregnancy significantly increases the volume of distribution of HCQ but does not affect the clearance or the 24 h area under the concentration–time curve. This model can be used to predict HCQ concentrations in pregnant women, helping healthcare providers to make informed decisions about dosing adjustments during pregnancy to ensure effective and safe treatment.

Etanercept is a biologic medication used to treat AIDs by inhibiting the activity of TNFα. A work by Yim and colleagues presented a popPK model of etanercept in pediatric patients with juvenile RA to identify PK parameters and their variability, and to explore the implications of once-weekly dosing compared to twice-weekly dosing [138]. The study used a popPK model developed from time–concentration data in pediatric patients, incorporating covariates including body surface area and sex on clearance, and body weight on the volume of distribution. The study concluded that the popPK model accurately predicted the PK profiles of etanercept in pediatric patients with RA, supporting the feasibility of a once-weekly dosing regimen for patient convenience.

A study by Chen et al. aimed to describe the PK of tacrolimus in children with SLE using real-world data [139]. The researchers developed a popPK model of tacrolimus, and they identified body weight as a significant covariate affecting the clearance and distribution volume in pediatric patients with SLE. Moreover, younger children tended to have a higher clearance of this drug. The popPK model can help in optimizing tacrolimus dosing in children with SLE, ensuring better treatment outcomes and minimizing side effects, such as nephrotoxicity, ototoxicity, and neurological adverse reactions. The purpose of the article of Petitcollin and colleagues was to develop a popPK model to detect and describe an early increase in infliximab clearance due to ADAs in children with CD, aiming to improve treatment outcomes by early detection of immunization to infliximab [140]. The proposed model was a time-varying clearance model that estimated the risk of developing ADAs, which increased linearly with time. This risk was logit-transformed to vary between 0 (no ADA influence) and 1 (full immunization effect). No significant covariates were identified for the parameters describing clearance modifications, such as baseline CRP and baseline PCDAI. However, the estimated value of clearance and infliximab trough concentration at week 2 were significant predictors of sustained remission. The model could detect an increase in infliximab clearance, allowing early detection of immunization to infliximab. This could help with dose adjustments in patients with CD and suggests that clearance variations could be used as a predictive marker of clinical response. This model may be used to optimize infliximab dosing regimens in children with CD by identifying patients at risk of immunization early, potentially improving treatment outcomes and reducing treatment failures [140].

A study by Zhou and coworkers aimed to predict the dose–exposure relationship of belimumab in Chinese pediatric patients with SLE using a popPK modeling approach as part of the drug registration process in China [141]. A linear two-compartment popPK model was initially built using data from adults, and then updated with pediatric data from the PLUTO clinical study to predict steady-state belimumab exposure in Chinese pediatric patients. The final model was a linear two-compartment popPK model that included both adult and pediatric data, which improved the prediction of belimumab’s PK in pediatric patients. Age was found to be a significant covariate affecting the volume of distribution in pediatric patients. Fat-free mass was another important covariate, affecting both clearance and volume of distribution, indicating that body composition plays a role in how the drug is eliminated and distributed. The results obtained indicated that the PK of belimumab was adequately described by the final model, supporting its use in predicting drug exposure in Chinese pediatric patients with SLE.

### 7.5. Other Autoimmune Disorders

The study of Romano-Aguilar et al. aimed to describe how MPA, a drug used to treat LN, behaves in the bodies of Mexican patients, and to identify covariates that explain variability in its PK [142]. The structural model was a two-compartment model with linear elimination. Creatinine clearance and the co-administration of prednisone were found to significantly influence the clearance of the drug, while body weight was found to influence the central volume of distribution of MPA. The study revealed that prednisone co-administration significantly increases the clearance of MPA, and this factor should be considered when prescribing MPA to optimize treatment for LN patients.

The study of Nader et al. aimed to evaluate the PK of upadacitinib in patients with UC and AD, and to support the E-R analyses of upadacitinib’s efficacy and safety in Phase II clinical trials for these diseases [143]. The study used popPK modeling and simulations to analyze data from multiple clinical trials, including Phase I and Phase II studies, to describe the PK of upadacitinib across different patient populations. A two-compartment model with first-order absorption for the immediate-release formulation and combined first- and zero-order absorption for the extended-release formulation was used to describe upadacitinib’s plasma concentration–time profiles. Significant covariates included creatinine clearance, disease state, and sex on apparent clearance, and sex and body weight on the volume of the central compartment. The study concluded that upadacitinib’s PK is consistent across different disease states and demographic groups, with no clinically relevant differences observed due to mild or moderate renal impairment, or between male and female subjects. This model can be used to predict upadacitinib exposure in different patient populations, aiding in dose selection and optimizing treatment regimens for UC and AD [143].

A study by Berends et al. aimed to evaluate the PK of adalimumab in patients with CD during induction and maintenance treatment, and to develop a new popPK model for treatment optimization [144]. The study used popPK modeling and external validation with independent datasets to evaluate the predictive performance of existing models, and to develop a new model using NLME modeling. The developed model was a one-compartment model describing the PK of adalimumab in patients with CD. This model estimated the clearance of adalimumab to be 0.32 L/day and the volume of distribution to be 4.07 L. The results of the study showed that the presence of ADAs significantly increased the clearance of adalimumab, and the newly developed model provided a better fit for the data compared to existing models. This model can be used to optimize individual dosing of adalimumab in patients with CD by considering significant covariates, thereby improving treatment outcomes and maintaining remission.

A study by Akpalu et al. aimed to assess the safety, tolerability, PK, PD, and immunogenicity of JNJ-61178104, a novel bispecific mAb against TNFα and IL-17 [145], in healthy subjects following single IV and SC administration [146]. PopPK modeling was used to describe the serum concentration–time data of JNJ-61178104 and to assess the effects of body weight and ADAs on its PK. A two-compartment model with first-order elimination was proposed, which included both administration routes. Body weight and ADA status were identified as significant covariates. Body weight affected the clearance and volume of distribution, while ADA status significantly increased the clearance rate of JNJ-61178104. This model can be used to predict the PK behavior of JNJ-61178104 in different populations, and to optimize dosing regimens by considering significant covariates.

A study by Suleiman and coworkers aimed to analyze the PK of risankizumab, an anti-IL-23 mAb, in healthy subjects and patients with moderate-to-severe plaque psoriasis using data from Phase I–III clinical trials [147]. An NLME modeling approach was used to analyze plasma PK data from 1899 subjects, including 13,123 observations. Simulations were carried out to evaluate the clinical relevance of covariates on risankizumab exposure, ensuring robust assessment by including parameter uncertainty. The PK of risankizumab was best described using a two-compartment model with first-order absorption and elimination. Risankizumab displayed linear PK across the evaluated doses, with no significant difference in exposure between healthy subjects and patients with psoriasis. This model can be used to predict risankizumab exposure in different patient populations, and to optimize dosing regimens for better therapeutic outcomes in treating moderate-to-severe plaque psoriasis.

A subsequent study by Suleiman and colleagues aimed to describe the PK of risankizumab in patients with psoriasis and CD [148]. The researchers used a two-compartment model with first-order absorption and elimination to describe the drug’s PK. Body weight and baseline albumin concentrations were the only significant covariates affecting risankizumab clearance. Body weight had a modest effect on drug exposure, while albumin had no meaningful impact. Risankizumab showed typical PK characteristics for an IgG1 mAb, with no significant differences between psoriasis and CD patients after accounting for body weight and albumin levels. This model can be used to predict risankizumab concentrations in different patient populations, helping to optimize dosing regimens in psoriasis and CD.

A study by Wojciechowski and coworkers aimed to develop and refine a popPK model for ritlecitinib, a JAK inhibitor tested for multiple autoimmune and inflammatory diseases, to support clinical drug development and inform dosing recommendations [149]. An iterative approach was used to develop three popPK models, incorporating new data at each stage to refine the model and address clinical questions. The final model was a two-compartment model with first-order absorption and direct-response non-stationary clearance and bioavailability driven by concentrations in the peripheral compartment. Body weight, inflammatory disease burden, and severe renal impairment were significant covariates affecting the clearance of ritlecitinib, with body weight being scaled allometrically and inflammatory disease burden consistent with other JAK inhibitors. Body weight and severe renal impairment were significant covariates affecting the volume of distribution. Dose, formulation (tablets or capsules), and high-fat meal effects were significant covariates affecting the absorption parameters of ritlecitinib. Moderate hepatic impairment was a significant covariate affecting the bioavailability of ritlecitinib.

An article by Morales et al. aimed to develop a quantitative DisP model for T1D to improve the identification of patient populations likely to progress to T1D within short-term clinical trial durations [60]. The study used a joint modeling approach that linked longitudinal glycemic measures to the timing of T1D diagnosis, incorporating baseline covariates through a stepwise covariate modeling approach and power functions. The proposed model was a joint DisP model that used a Weibull model to capture the timing to T1D diagnosis and a sigmoid Emax function to quantify 2 h oral glucose tolerance test (OGTT) values as a time-varying biomarker. Significant covariates included baseline HbA1c and the presence of different autoantibodies (AAbs), such as GADA, which were associated with parameters representing DisP. The developed T1D DisP model accurately reflected data from TEDDY and PTP natural history clinical studies, and it was validated using internal hold-out and the TN10 dataset. This model can be used in future clinical trial simulations to optimize trial design, including selecting biomarkers, determining inclusion/exclusion criteria, and estimating the optimal number of participants and trial duration [60].

Eculizumab is used to treat generalized MG by inhibiting the terminal complement C5. The study of Monteleone et al. tested one- and two-compartment popPK models to analyze PK data obtained from MG patients treated with eculizumab, and it investigated the impact of various covariates on PK parameters using NLME modeling [150]. Body weight and plasma-exchange events were significant covariates affecting the clearance of eculizumab. In addition, the researchers performed PK/PD analysis using biomarkers including free C5 concentration and in vitro hemolytic activity, which helped in assessing the drug’s efficacy in inhibiting terminal complement activation. The study concluded that the approved dosing regimen of eculizumab rapidly achieves and maintains complete inhibition of terminal complement activation, providing sustained clinical efficacy in patients with generalized MG. This model could be used in the future to optimize dosing regimens.

The study of Wendt and coworkers aimed to create a reliable model to describe how glucagon affects glucose production in patients with T1D, which can help in preventing or treating low blood sugar and improving artificial pancreas systems [151]. The researchers used PK and PD models to simulate the effects of insulin and glucagon on glucose levels, validated through leave-one-out cross-validation. The proposed model included equations and parameters to simulate glucose excursions based on plasma insulin and glucagon concentrations, incorporating effects of both hormones on endogenous glucose production. The study concluded that the PD model accurately simulates glucose levels, making it useful for in silico simulations to improve diabetes treatment strategies.

A study by Sokolov and coworkers aimed to identify factors that define the response of short- and long-term glycemic markers to dapagliflozin when used as an add-on to insulin therapy in T1D [152]. The study adapted a mechanistic model of type 2 diabetes mellitus for T1D by replacing endogenous insulin with exogenous insulin and introducing insulin-dependent feedback on glucose production. The data included plasma glucose levels and HbA1c responses to dapagliflozin, analyzed using simulations and modeling techniques. The study found that decreasing the insulin dose by 15–38% can counterbalance the average glucose reduction by dapagliflozin treatment. The developed platform serves as a quantitative tool for in silico trials of combined dapagliflozin–insulin treatment, aiding in optimal dose selection and understanding the physiological system in T1D.

The purpose of the article of Chen et al. was to develop a popPK/PD model for tacrolimus in patients with MG to understand the relationships among tacrolimus dose, exposure, and therapeutic efficacy [153]. Simulations were performed to assess the impact of different covariates on the PK profiles and therapeutic efficacy. The proposed model described the relationship between the cumulative area under the curve of tacrolimus and the quantitative MG scores using an Emax function. Significant covariates included the CYP3A5 genotype, which influenced the apparent clearance, and total protein, which impacted the apparent volume of distribution. Osserman’s classification was also a significant covariate on the initial score of patients with MG. The primary PD marker used was the total quantitative score of MG, which was treated as a continuous variable in the proposed PK/PD model. The study concluded that tacrolimus showed an unsatisfactory effect in some patients due to insufficient exposure, and higher doses might be required for patients with certain CYP3A5 genotypes and lower TP levels to achieve rapid therapeutic action.

Table 5 presents a summary of population modeling approaches in clinical applications and the development of new medications for the treatment of AIDs.

Pharmacometrics is integral not only to pre-clinical drug discovery and development, but primarily in clinical settings by enabling precise prediction of drug behavior, efficacy, and safety across diverse populations and individuals. Through popPK, popPK/PD, and population-based DisP models, pharmacometrics facilitates the identification of key covariates that influence drug disposition and response. These models aid in optimizing dosing regimens, predicting therapeutic outcomes, and addressing variability in drug responses in complex diseases, such as AIDs. However, this approach is much more frequently used in clinical applications in developed countries compared to low- and middle-income nations. In AIDs, it is utilized to optimize treatment in order to quickly achieve and maintain remission, increase the safety of therapies, and to avoid flares of these diseases, thereby substantially reducing the costs of treatments.

## 8. PBPK Modeling in Autoimmune Diseases

Treating AIDs presents several challenges, particularly regarding drug interactions and the impact of inflammation on drug PK. Inflammation and organ dysfunctions, which are present in many AIDs, can alter drug absorption, distribution, metabolism, and excretion. Interactions of drugs with endogenous molecules are also difficult to predict and may alter treatment outcomes. Additionally, patients with AIDs often require multiple medications, increasing the risk of harmful drug interactions. This complexity makes it essential to carefully monitor treatment plans and adjust dosages to optimize efficacy while minimizing adverse effects. PBPK modeling is capable of predicting drug kinetics by taking into account changes in blood flow through the organs, changes in the activity of metabolizing enzymes, drug interactions, the disease state of organs (such as liver and kidney impairment), and other characteristics of diseased individuals.

The study of Tse and colleagues aimed to predict how drug interactions and kidney or liver dysfunctions affect the PK of tofacitinib by utilizing a PBPK model [156]. The researchers used the Simcyp software version 15.1 (Simcyp Ltd., Sheffield, UK, a subsidiary of Certara LLC) to create the PBPK model based on the physical and chemical properties of tofacitinib, as well as data from pre-clinical and clinical studies. They verified the model by comparing its predictions with actual clinical data, including how the drug behaves when taken alone or with other drugs that affect liver enzymes. The study found that tofacitinib’s PK can be affected by other drugs that inhibit or induce liver enzymes, including CYP3A4 and CYP2C19. The main conclusion was that the PBPK model is a reliable tool for predicting how tofacitinib behaves under different conditions, such as kidney or liver impairment and drug interactions, reducing the need for additional clinical studies.

Sirukumab is an anti-IL-6 mAb used in patients with RA. The purpose of the article of Jiang et al. was to develop a PBPK model to predict the impact of elevated IL-6 levels on CYP enzymes and the treatment effect of sirukumab in RA patients [157]. The study used modeling and simulation strategies to predict the PK of multiple CYP substrates in RA patients before and after sirukumab treatment. The proposed model was a PBPK model that incorporated the impact of systemic IL-6 levels on the expression of multiple CYP enzymes in the liver and intestines. It used in vitro data to simulate the modulation of the CYP enzymes by IL-6 and the effects of sirukumab treatment on these enzymes. The PBPK model successfully captured the modulation effect of IL-6 and sirukumab on the activity of CYP3A, CYP2C9, CYP2C19, and CYP1A2. This model can be used to predict the PK of small-molecule drugs in RA patients and assess the impact of anti-IL-6 treatment. The model could be used in the future to explore the impact of RA and anti-IL-6 treatment on the metabolism and PK of other small molecules metabolized by CYP enzymes. It can also serve as a framework for developing PBPK models for other cytokine-neutralizing antibodies.

The aim of the article of Machavaram et al. was to explore the impact of IL-6 on the suppression of drug-metabolizing enzymes, particularly CYP3A4, and to predict the clinical drug–drug interactions between therapeutic proteins and small-molecule drugs in disease settings associated with elevated cytokine levels, such as AIDs [158]. The study used in vitro–in vivo extrapolation and PBPK models to simulate the PK of simvastatin and cyclosporine in patients with elevated IL-6 levels due to inflammatory conditions, such as RA. Elevated IL-6 levels significantly suppress CYP3A4 activity, leading to altered drug metabolism and potential drug–drug interactions. This model may be used in the future to predict drug–drug interactions in other inflammatory diseases and AIDs, to guide dose adjustments of co-administered drugs in patients with elevated cytokine levels.

HCQ is used for treating RA, SLE, and other diseases. The purpose of the article by Alqahtani et al. was to predict the PK of HCQ in healthy individuals and extrapolate these predictions to populations with liver cirrhosis and chronic kidney disease using a PBPK model [159]. The proposed model was a whole-body PBPK model designed to simulate HCQ PK in healthy individuals and extrapolate it to liver cirrhosis and chronic kidney disease populations by incorporating disease-specific pathophysiological changes. The PBPK model was able to predict HCQ PK in healthy individuals and diseased populations, showing increased drug exposure in liver cirrhosis and decreased AUC in chronic kidney disease. This model may be used to design optimal dosing regimens for HCQ in patients with varying degrees of liver and kidney impairments.

A study of Wang et al. aimed to expand a PBPK model to assess potential therapeutic protein–drug interactions between anti-IL-6 treatment and CYP substrate drugs in various immune-mediated disease populations with elevated IL-6 levels [160]. A PBPK model was utilized to simulate the impact of elevated IL-6 levels and anti-IL-6 treatment on multiple CYP enzymes. The study analyzed the modulation effects of IL-6 on CYP enzymes using simulations at different hypothetical IL-6 levels. The study concluded that elevated IL-6 levels significantly modulate CYP enzyme activity, and that anti-IL-6 treatment can alter this modulation. The PBPK model successfully predicted the interactions in various populations, providing insights into the potential impact of IL-6 on drug metabolism.

Aspirin-triggered resolvin D1 (AT-RvD1) is known for its potential to reduce inflammation and restore tissue integrity in the salivary glands of patients affected by SjS. The aim of the article of Yellepeddi and coworkers was to create a PBPK model to determine the appropriate dosage of AT-RvD1 for future studies in mice and humans, aiming to reduce the need for excessive use of animals and humans in trials [161]. The study employed modeling software. such as PK-Sim and MoBi version 7.4 to predict the PK of AT-RvD1 in both plasma and saliva following IV administration in NODShiLtJ mice. The proposed model described the PK of AT-RvD1 in NODShiLtJ mice and humans, predicting how the drug was distributed and eliminated from the body. The results indicated that AT-RvD1 follows a one-compartment PK pattern with a long elimination half-life and rapid distribution into highly perfused tissues. It was concluded that PBPK modeling can improve the targeting of in vivo studies and has the potential to enhance drug development and clinical trial design.

A study by Sharma et al. aimed to explore whether a PBPK model could be combined with a relative transcriptomics approach to predict local anifrolumab tissue concentrations and receptor occupancy [162]. The data analyzed included demographic information from clinical studies, dosing regimens, and virtual trial simulations. Anifrolumab at 120 mg administered SC every week was found to be equivalent to 300 mg IV every four weeks in achieving high local concentrations and receptor occupancy. The study concluded that the PBPK model could inform dosing for Phase III trials in new indications, providing a novel approach to target expression optimization in clinical development.

A study by Effinger and colleagues aimed to develop an in vitro biorelevant dissolution methodology and a PBPK model to predict the performance of budesonide in healthy subjects and CD patients [163]. The researchers used in vitro biorelevant dissolution tests and PBPK modeling to simulate the release of budesonide from the controlled-release formulation Entocort^®^ under healthy and CD conditions. The study successfully predicted budesonide exposure after IV and oral administration using the developed PBPK model. It found that CD patients have higher budesonide exposure compared to healthy subjects. The study concluded that PBPK modeling combined with in vitro release testing can predict changes in drug bioavailability due to CD, guiding dosage adjustments for CD patients.

The discussed examples of PBPK model applications in drug development and therapy optimization are listed in Table 6.

PBPK modeling is a valuable tool for understanding and predicting drug interactions and the impact of organ impairments on drug behavior. These models enable the simulation of complex scenarios, such as drug–drug interactions and the impact of disease-specific inflammatory mediators on drug PK. In addition, they help to optimize dosing regimens, tailor treatments to individual patient needs, and predict the occurrence of adverse reactions, thereby improving the safety and efficacy of therapies for AIDs.

## 9. QSP in Autoimmune Diseases

QSP modeling is relatively new and highly mechanistic approach compared to the PK/PD models previously discussed. While conventional PK/PD models primarily focus on the relationships between drug concentrations and their effects, often using simplified or empirical representations, QSP integrates detailed biological pathways and systems biology into the modeling process.

One of the examples of QSP’s application is presented in the article of Coto-Segura and colleagues, who developed and validated models to describe the PK and PD of certolizumab pegol in patients with moderate-to-severe psoriasis, aiming to improve personalized treatment strategies [164]. Certolizumab pegol is a PEGylated antigen-binding fragment of a humanized mAb against TNFα. The researchers used PBPK and systems biology (SB) models to simulate the drug’s PK and its effects on psoriasis-related proteins. The proposed approach integrated PBPK and SB models to create QSP models. These models enabled the simulation of drug concentrations in different organs and the assessment of their impact on disease-related proteins, using a two-compartment system for skin and blood. Significant covariates included cardiac frequency, age, BMI, and sex, which influenced drug distribution and clearance. PD markers included inflammatory proteins such as IL1β, TNFα, and CRP, as well as proteins related to skin immunological barriers, angiogenesis, and metabolism, which were used to measure disease severity and treatment efficacy. The study’s results revealed that the QSP models could accurately predict clinical and molecular efficacy features of certolizumab pegol, supporting their use in generating hypotheses for personalized treatment in psoriasis patients. These models could be used in the future to tailor treatment plans for individual patients, predict drug efficacy in diverse subpopulations, and improve the understanding of the molecular mechanisms underlying psoriasis and its treatment [164].

Recombinant human IL-10 (rhuIL10) was tested for its therapeutic efficacy in CD. The aim of the study of Balbas-Martinez et al. was to develop a QSP model to describe the dynamics of ILs in CD and to evaluate potential therapeutic strategies using this model [165]. The pharmacometric approaches used included QSP modeling, sensitivity analysis, and simulation of therapeutic interventions using the SimBiology toolbox in MATLAB. The QSP model characterized the dynamic behavior of plasma ILs in CD, integrating data from the literature to simulate the effects of IL-based therapies. The PD markers used in the study included IL-10, IL-6, IL-1β, and others, which were quantified to evaluate the therapeutic effects of rhuIL-10. The QSP model effectively simulated the dynamics of ILs in CD. This model could be also employed to evaluate potential therapeutic strategies, identify new therapeutic targets, evaluate the efficacy of different drug combinations, and simulate clinical scenarios to improve treatment strategies for CD.

The purpose of the work of Hurez et al. was to develop a QSP model for SjS that links mechanistic signaling pathways to clinical outcomes, aiming to evaluate novel drugs for SjS and optimize clinical trial outcomes [166]. The mechanistic QSP model focused on salivary gland and lymph node manifestations in SjS, incorporating the PK of standard-of-care drugs in the blood, salivary glands, and lymph nodes. The proposed model was the SjS PhysioPD research platform, which includes compartments for salivary glands, lymph nodes, and blood, with relevant cell types such as gland epithelial cells, T and B lymphocytes, and antigen-presenting cells, regulated by cytokines and chemokines. The developed platform could predict complex clinical scores from mechanistic outputs, demonstrating the predictive value of QSP models in drug development and facilitating their adoption by clinical teams.

A study by Rogers and colleagues aimed to develop a mechanistic, multistate, QSP model of inflammatory bowel diseases (CD and UC) to explore treatment mechanisms and aid in clinical drug development. The model was created by incorporating key biological mechanisms, including cell differentiation and cytokine production. The simulations of the model for clinical outcomes, CRP, and fecal calprotectin were consistent with published data [167].

A subsequent study by the same research group aimed to evaluate the performance of the QSP model by using clinically observed biomarker changes following different treatments [168]. The researchers used the model to simulate multiple therapies and compare published clinical outcomes. They also created and tested hypotheses with treatment biomarker responders and predicted the biomarker responses for combination therapies in silico. The data were sourced from the literature as well as in-house data. The study provided insights into the behavior and variability of new therapies and combination treatments in inflammatory bowel diseases. It also offered clues as to mechanistic reasons behind the failure of previous treatment modalities [168].

As shown in this section, QSP models play an important role in advancing the treatment of AIDs by integrating complex biological pathways with drug PK and PD to predict treatment outcomes and optimize therapeutic strategies. QSP models, such as those developed for certolizumab pegol in psoriasis and recombinant human IL-10 in CD, simulate drug interactions within the body and their effects on disease-related proteins, allowing for personalized treatment plans and hypothesis generation for novel therapies [165]. The QSP model for primary SjS links mechanistic pathways to clinical outcomes, facilitating the evaluation of novel drugs and optimizing clinical trial designs [166]. Additionally, systems pharmacology models help to identify drug targets, biomarkers, and patient subpopulations, improving the precision and success of therapeutic interventions. These models are especially important in understanding the molecular mechanisms of AIDs, predicting drug efficacy in diverse patient populations, and tailoring treatments to individual patient needs.

## 10. Boolean Network Modeling in Autoimmune Diseases

Boolean networks offer a simplified yet powerful framework to model complex biological systems by representing components in binary states. They are widely used to explore dynamic interactions in gene regulatory networks, signal transduction pathways, and disease mechanisms, providing valuable insights into system behavior and therapeutic targets [10,169].

Ruiz-Cerdá and coworkers proposed Boolean networks to study the processes involved in antigen presentation in SLE, aiming to support drug development by identifying targets, biomarkers, and patient subpopulations [58]. The Boolean network model, which is less data-demanding and can provide insights into the dynamics of biological networks, described the immune responses to autoantigens in SLE. It included nodes representing different biological components and their interactions, including activation, inhibition, upregulation, or downregulation. The researchers concluded that the presented model could help to identify drug targets, optimal drug combinations, and patient subpopulations, potentially improving the success rate of clinical trials by enabling patient stratification [58].

A research paper by Miagoux and colleagues aimed to unravel the mechanisms governing the regulation of key transcription factors in RA and derive patient-specific models to understand disease heterogeneity and treatment response [170]. The researchers used a systems biology approach, integrating transcriptomics datasets and ML to create an RA-specific transcription factor co-regulatory network. They further enriched this network with signaling cascades and upstream regulators using a molecular map specific to RA. The study identified master regulators and upstream cascades affected by anti-TNF treatment. It also simulated network subparts to identify conditions that can switch transcription factors on or off, mimicking the effects of perturbations [170].

A study by Kennedy et al. aimed to explore the complex nature of MS by analyzing the disease across multiple biological scales, from genes and proteins to cells and tissues, up to the full organism [171]. The researchers utilized multilayer network analysis and deep phenotyping with multi-omics data, brain and retinal imaging, and clinical data to understand the interplay among these scales in MS. Boolean networks were used in the study to analyze the complex interactions within the biological systems related to MS. Boolean simulations were employed to provide a qualitative description of the system by considering nodes in one of two states: active or inactive. These simulations helped identify paths among different biological layers, connecting molecular aspects with clinical phenotypes. This research highlights the potential of using multilayer network analysis to better understand complex diseases like MS, and to identify specific biological pathways that contribute to disease manifestation [171].

## 11. Model-Based Meta-Analysis in Autoimmune Diseases

MBMA is a method that integrates published summary data with internal datasets to support critical decision-making throughout the drug development process. This approach is especially useful in evaluating the benefit–risk profile of investigational treatments. MBMA facilitates the identification of optimal dosing strategies by incorporating data from historical external comparators for specific disease indications, including AIDs. Its flexibility in synthesizing and interpreting aggregated data makes it an important component of pharmacometrics, enabling more informed evaluations of various therapeutics [11].

An article by Chen et al. aimed to quantitatively evaluate the placebo effect as well as the efficacy of eculizumab and efgartigimod in treating MG by analyzing factors affecting the quantitative MG score and the MG activities of daily living score [172]. MBMA was used, which involved a search of randomized placebo-controlled clinical trials in public databases, such as PubMed, EMBASE, and Cochrane Library. A PK/PD model was developed to describe the time-course of drug efficacy and the placebo effect, using data from 12 articles including 13 trials with 673 participants. Eculizumab showed the highest efficacy in reducing MG scores by 3.66 points, while efgartigimod was most effective in reducing the MG activities of daily living scores, by 1.97 points. The placebo effect increased over time, reaching significant levels in 12 weeks. The study provided quantitative information crucial for MG drug development and highlighted the impact of thymectomy on daily living activities [172].

A research article by Chan et al. aimed to examine the clinical efficacy data of fenebrutinib in patients with RA [96]. The study utilized MBMA using Phase II clinical trial data for fenebrutinib. Additionally, a summary-level clinical efficacy database was constructed using data from the literature. MBMA confirmed the consistency of ACR20, ACR50, and ACR70 responder rates with historical data. This approach maximizes knowledge extraction from the efficacy data.

Wang and coworkers aimed to assess the relationship between short-term and long-term treatment effects in RA, specifically using ACR50 responses, and to evaluate the feasibility of predicting 6-month efficacy from short-term data [173]. A generalized non-linear model was developed to quantify the relationship between 3- and 6-month ACR50 treatment effects, and to test the impact of covariates. The analysis used an RA database constructed from 68 reported trials, representing data from over 27,000 patients treated with 13 different drug classes. The study found a strong correlation between 3-month and 6-month ACR50 treatment effects, with a scaling factor close to 1, indicating that 3-month effects were approaching a plateau. The findings support using 3-month efficacy data to predict long-term efficacy, suggesting that early efficacy readouts can inform the probability of clinical success [173].

An article by Demin et al. aimed to demonstrate how a quantitative assessment of the longitudinal time-course of the clinical efficacy of therapeutic agents for RA can support the development of novel biologics during early clinical development [174]. A longitudinal MBMA was employed to assess the magnitude and time-course of treatment responses. The analysis included data from 37 double-blind Phase II–III randomized clinical trials in adult RA patients. The data were extracted from published sources, including PubMed and FDA drug labeling information. The study found that capturing the longitudinal components of drug effects allows for better indirect comparisons of drug responses [174].

The study of Goteti et al. aimed to benchmark SLE treatment effects and identify clinically important covariates using MBMA for efficacy endpoints and SLE composite endpoint scores [175]. A Bayesian MBMA framework was employed, utilizing a latent variable model to describe aggregate SLE endpoints over time for various placebo and treatment arms. Continuous dose–effect relationships were modeled for specific treatments, while others were treated as discrete dose effects. The dataset consisted of 25 studies and 81 treatment arms evaluating 16 different agents. Data were sourced from PubMed and clinicaltrials.gov, focusing on study and investigational agent, dose and regimen, baseline descriptors, and outcomes. The study illustrated the application of latent variable models in understanding chronic disease trajectories and aimed to enable MIDD of new drug candidates in SLE [175].

The study of Sui et al. aimed to conduct an analysis and comparison of the efficacy of different drugs against primary progressive MS using an MBMA approach [176]. The data analyzed included 15 studies involving 3779 patients, sourced from the PubMed, EMBASE, and Cochrane Library databases. These studies included nine placebo-controlled and six single-arm trials. The study found that nine out of twelve drugs showed significantly better efficacy than placebo, with ocrelizumab demonstrating outstanding performance. This study provided necessary quantitative information for the rational clinical use of drugs and future clinical trials in primary progressive MS.

The study of Liu et al. aimed to develop an E-R model for secukinumab to recommend dose regimens for patients with moderate-to-severe plaque psoriasis based on different body weights [177]. An MBMA was conducted using data from randomized controlled trials, focusing on the PASI 75 and PASI 90 response rates as primary outcomes. Data from 16 trials involving 6197 subjects were analyzed. The data were sourced from the PubMed and Cochrane Library databases. The model accurately described the response rates over 52 weeks. It recommended different maintenance doses based on patient body weight, suggesting more economical and efficacious regimens for patients with moderate-to-severe plaque psoriasis.

The article of Dodds et al. aimed to explore MIDD as an alternative to traditional empirical methods, focusing on improving the efficiency of clinical trials and drug development processes [178]. The researchers utilized MBMA to pool data across studies, characterizing dose–response relationships and defining relationships between biomarkers and efficacy endpoints using quantitative models. Data for the MBMA were sourced from literature searches, previous meta-analyses, clinicaltrials.gov, conference abstracts, and corporate websites. The data included 27 trials involving anti-TNF, ustekinumab, and methotrexate. The study demonstrated that model-based approaches could enhance the design and decision-making processes in drug development, particularly in making Go/No-Go decisions and dose–response learning. It highlighted the potential of distributed trial designs to provide valuable dose–response information, albeit with a slight decrease in decision-making accuracy [178].

The study of Maloney et al. aimed to compare 16 drugs for moderate-to-severe PA using an MBMA approach based on the ACR response criteria [179]. Data from 49 trials involving 16 drugs and a total of 21,340 patients were analyzed that were sourced from a search of randomized clinical trial data from Medline, ClinicalTrials.gov, FDA documents, and sponsor websites. The study identified a predictive MBMA model that accurately predicted trial outcomes 90% of the time. The model described changes in ACR20/50/70 over time as a function of various factors, including drug and dose. This approach provided a comprehensive comparison of treatments and could be used for calculating the probability of success in future trials.

Dupilumab is an mAb that blocks the activity of IL-4 and IL-13 [180]. An article by Miyano and colleagues aimed to understand the variability in drug response among patients with AD, particularly focusing on those who responded poorly to dupilumab. It sought to identify promising drug targets for these poor responders [181]. An MBMA of recent clinical trials involving nine biologic drugs for AD, including dupilumab, lebrikizumab, tralokinumab, secukinumab, fezakinumab, nemolizumab, tezepelumab, GBR 830, and recombinant IFN-γ, was conducted. A mathematical model was developed to simulate the clinical efficacy of hypothetical therapies on virtual patients. The model successfully reproduced the reported time-courses of improved EASI and EASI-75 for the nine drugs. This suggests that this model can be used to simulate and predict the efficacy of potential therapies for poor responders to dupilumab in AtD [181]. A summary of MBMA applications in advancing therapies for AIDs is presented in Table 7.

## 12. Incorporating AI and ML into Pharmacometrics in the Context of Autoimmune Diseases

The integration of AI and ML algorithms has, in many cases, significantly advanced pharmacometrics. However, the utility of these approaches is at an early stage of development. ML methods can complement pharmacometrics by efficiently identifying important variables and their interdependencies, enhancing predictive capabilities and learning from large datasets. ML algorithms may constitute a computational bridge between big data and population modeling, enabling better parameter inference and insights into drug effects across diverse populations. However, some ML and AI techniques, such as neural networks, are perceived as black boxes, making it difficult to extract mechanistic insights from these models. Neural networks have been shown to perform poorly with sparse and noisy data. Therefore, there is a need to identify reliable and robust ML and AI workflows for each pharmacometric application to ensure that the findings are generalizable. Nonetheless, ML algorithms are computationally efficient and can process large datasets, enabling learning and hypothesis generation from big data, which are crucial in drug development. In addition, ML facilitates model building and simulation, which can accelerate drug development and improve patient outcomes. Moreover, the integration of ML and AI offers innovative quantitative methods to extract insights from new data, addressing unmet needs in pharmacometrics [182,183,184]. This section presents specific examples of how AI and ML algorithms have been employed to advance pharmacometric methodologies for treating AIDs, as well as a few cases of their broader applications in pharmacometrics beyond autoimmune conditions.

A study by Turner aimed to evaluate different Natural Language Processing (NLP) classifiers for recognizing SLE patients from electronic health records, and to compare them with a novel Bayesian word vector approach [185]. The researchers applied multiple NLP and ML methods, including naïve Bayes, neural networks, random forests, support vector machines (SVMs), and Word2Vec inversion. These models were implemented using Python libraries. The findings indicated that neural networks using Concept Unique Identifiers (CUIs) and random forests with either Bag of Words or CUIs achieved comparable performance in classifying unstructured clinical notes. New methodologies can be more easily scalable beyond binary classification tasks and require less overhead compared to traditional methods.

A study conducted by Adamichou and colleagues aimed to develop a diagnostic model for SLE. The researchers applied ML techniques, specifically random forests and LASSO-logistic regression, to analyze data from 802 adults diagnosed with either SLE or other rheumatological conditions. Statistical analyses were conducted using R software version 3.5.1, SPSS version 25.0, and MATLAB version 9.2. The model incorporated 14 weighted clinical and serological features, designed to categorize patients by the possibility of suffering from SLE. This model demonstrated excellent sensitivity and specificity in diagnosing SLE, effectively identifying both early and severe forms, and showing promise as a valuable diagnostic tool in clinical settings [186].

A study by Cheheltani aimed to develop an ML model to identify T1D patients who were incorrectly diagnosed with type 2 diabetes, and to examine the risk factors associated with such misdiagnoses [187]. Using de-identified electronic medical records from the IQVIA AEMR database, which includes data from over 100 thousand physicians across all US states, the researchers analyzed 932 variables. Through recursive feature elimination, these were refined to 250 key variables. The results indicated that misdiagnosed patients were generally younger and had lower weight and BMI. This algorithm demonstrated the ability to be integrated into EMR systems as a support tool, revealing complex associations within patient histories. Such a tool could aid in lowering misdiagnosis rates and promoting more effective early-stage care strategies. The study found an estimated 10% misdiagnosis rate among its cohorts, which could be a conservative figure.

A study by Guo aimed to assess the role of metabolism-related genes in the development of RA, and to identify potential diagnostic biomarkers [188]. The researchers employed a range of methods, such as NMR spectroscopy for metabolite analysis, PCR for biomarker validation, and single-sample gene set enrichment analysis to analyze immune infiltration. AI and ML algorithms played critical roles throughout the study, helping refine the selection of diagnostic biomarkers. Specifically, the Least Absolute Shrinkage and Selection Operator algorithm identified 56 potential biomarkers, while the random forest algorithm further refined the selection to 14 diagnostic markers. By combining Weighted Gene Co-Expression Network Analysis, Least Absolute Shrinkage and Selection Operator, and random forest algorithms, the researchers narrowed down to the four primary biomarkers, including AKR1C3, MCEE, POLE4, and PFKM. The study identified four MRGs as diagnostic biomarkers for RA and established their correlation with immune infiltration patterns.

An article by Basu et al. aimed to develop a predictive modeling approach to identify patients with MS who will experience disease activity in 3 and 6 months, using ML methods to analyze clinical covariates and improve prediction accuracy [189]. The researchers used supervised ML models, specifically XGBoost, to learn the relationships between high-dimensional input covariates and the output variable, predicting disease activity at 3- and 6-month intervals. The model used was XGBoost, an ensemble tree-boosting method, combined with Shapley Additive Explanations (SHAP), to make the decision process transparent. XGBoost handles multicollinearity well and does not require covariate selection, while SHAP explains the contribution of each covariate to the model predictions. The study concluded that using complex ML models like XGBoost combined with explainability methods like SHAP can effectively analyze high-dimensional data, providing interpretable insights into MS DisP and aiding in clinical decision-making. This model can be used to plan treatment, decide the frequency of patient visits, and prompt other clinical interventions by predicting future disease activity in MS patients, thus supporting clinical decision-making.

A study conducted by Yao and colleagues aimed to identify biomarkers that could help in diagnosing psoriasis and understanding its association with the pathophysiology of the disease [190]. Gene expression data were obtained from the Gene Expression Omnibus (GEO) database. Differentially expressed genes (DEGs) were used to create volcano plots using R ggplot2 V3.3.5, heat maps with Pheatmap V1.0.12, and Venn diagrams with Venn Diagram V1.6.20. Functional enrichment analysis was then performed using the Goplot V1.0.2 package and the clusterProfiler V3.18.1 software. To identify key psoriasis signatures and potential biomarkers, four different algorithms were applied. The associations between selected biomarkers and immune cell populations were examined using Spearman’s correlation. In conclusion, the researchers identified the *ADAM23* gene as a key biomarker, as it appeared in all analyzed critical signature algorithms. Additionally, CIBERSORT analyses showed its association with mast cells and macrophages. All of these findings suggest that ADAM23 could be a potential biomarker for diagnosing patients with psoriasis.

The purpose of the work of Myers et al. was to present a novel workflow that uses ML surrogate models to improve the efficiency of generating virtual patients for QSP models, which may help in exploring variability and uncertainty in clinical responses [191]. The researchers used QSP modeling and ML surrogate models to generate virtual patients more efficiently by pre-screening parameter combinations that resulted in feasible virtual patients. The proposed model involved training ML models using the full QSP model and then using these surrogate models to rapidly pre-screen parameter combinations for generating valid virtual patients. The main conclusion was that ML surrogate models significantly improved the efficiency of generating virtual patients for QSP models, which can enhance the utility of QSP research across the drug development pipeline.

An article by Stankevičiūtė and coworkers discussed various pharmacometric approaches, including PK and PD modeling, popPK, and model-informed precision dosing [184]. The authors proposed integrating ML with traditional pharmacometric models, such as using deep neural networks combined with ODEs to improve NLME modeling for predicting drug doses and simulating treatments. ML could help determine the best exposure indices and their relationship to microscopic targets or macroscopic outcomes. The main conclusion was that combining the strengths of pharmacometric modeling with the flexibility of ML can lead to better individualized treatment plans, improving the risk–benefit balance for patients.

In turn, Zhang et al. aimed to explore how combining QSP and ML can improve drug development by addressing key questions, such as the mechanism of action of drugs and DisP [192]. The methods involved analyzing multilayer ”omics” data, such as gene expression and proteomics, using ML approaches, and integrating these with QSP modeling to overcome the limitations of each method.

The integration of QSP and ML is still in its early stages in treatment advancement for AIDs, with several successful implementations showing promise in improving decision-making in drug development processes. Future research will focus on evaluating available technical tools to better integrate QSP and ML for more effective drug development. For instance, ML can be used to select the most important features from large datasets, which can then be used to develop more focused QSP models that include only the highly relevant features. QSP models establish a framework for identifying the most informative data for scientific discovery, requiring an iterative workflow to generate new data and refine the models continuously.

The incorporation of AI and ML into pharmacometrics represents a transformative approach, especially in the context of AIDs. By leveraging the strengths of ML, such as handling high-dimensional data and complex relationships, this approach can significantly enhance predictive modeling and decision-making processes [193]. For example, ML models like XGBoost, combined with interpretability tools such as SHAP, have been used to predict disease activity in MS, offering clinicians valuable insights into patient-specific disease trajectories and enabling more tailored treatment strategies [189]. In QSP models, ML aids in efficiently generating virtual patients by using surrogate models, which accelerate the exploration of variability and uncertainty in clinical responses [191]. Moreover, integrating ML with traditional pharmacometric approaches, such as NLME modeling, can refine dose prediction and optimize treatment regimens [184]. Additionally, the ability of ML to analyze multilayer “omics” data and identify key biomarkers may enhance our understanding of disease mechanisms and support the development of more effective therapies [192]. The integration of pharmacometrics and ML may enable the development of more focused and mechanistically informed models that will enhance the predictive power and overall efficiency of pharmacometric analyses. As AI and ML continue to evolve, their role in pharmacometrics is expected to grow, driving innovations in how we understand, predict, and treat complex diseases such as AIDs.

## 13. Limitations of Pharmacometric Methods

The implementation of pharmacometric methods in scientific research and clinical practice faces several challenges and limitations. For instance, PBPK models require extensive input data, including tissue-specific drug binding and enzyme/transporter expression levels [6]. Inadequate or inaccurate data can lead to unreliable predictions. Complex models, especially those with many parameters (e.g., QSP, PBPK, and PK/PD models), can be difficult to validate comprehensively. Even if the model fits the data well, understanding its mechanistic soundness and identifying sources of error can be challenging [194]. PBPK models often aim to predict drug behavior across diverse populations (e.g., pediatric, elderly, or specific disease states). Validating these predictions is difficult due to the scarcity of clinical data for many subpopulations [195].

PK/PD models are frequently used to predict long-term outcomes based on short-term data. Validation and verification of such models requires longitudinal studies, which are time-consuming and expensive, limiting their availability. Simple empirical PK/PD or compartmental PK models fail to capture the underlying biological processes, limiting their predictive power for novel scenarios. Extrapolation beyond studied conditions (e.g., different dosing regimens or populations) can lead to inaccuracies [196]. DisP modeling also requires longitudinal clinical data, which may be sparse or incomplete. These models are often described by simple algebraic functions and might not capture the full complexity of disease mechanisms, especially in multifactorial diseases such as AIDs [197].

In population-based models, high computational demands and difficulty in convergence during model fitting can hinder their practical use. While population models aim to capture variability, ensuring their validity across different demographics and clinical settings is difficult. Sparse data from underrepresented groups (e.g., specific ethnicities) can compromise model performance [198].

QSP models require extensive biological and pharmacological data, leading to high computational costs and increased risk of overparameterization. Incomplete knowledge of biological pathways and insufficient experimental data can reduce model accuracy [199]. QSP models are very complex, making them difficult to validate and less accepted by regulatory agencies for decision-making. QSP models aim to provide mechanistic insights at multiple biological levels. However, experimental validation of these predictions is often not feasible due to technical or ethical limitations, especially in humans. QSP models often include hundreds of parameters representing complex biological networks. Validating all components comprehensively is frequently unfeasible. Moreover, errors in one part of the model may propagate, affecting the overall predictions [199,200]. On the other hand, the binary nature of Boolean networks oversimplifies biological processes, which often operate on a continuum. These models are more suitable for qualitative insights and lack the precision needed for dose–response predictions. In addition, Boolean models require manual tuning or heuristic methods, which can be subjective and non-robust [169,201].

MBMA combines data from multiple studies to inform drug development decisions. Variability in study design, patient populations, and endpoints can introduce biases and reduce model reliability. MBMA depends on published data, which may overrepresent studies with positive outcomes [11,202].

In clinical practice, pharmacometric models need to be accepted by regulatory agencies, such as the FDA or EMA. The approval process can be slow, especially when novel methods are involved. Ethical concerns may also arise when using pharmacometrics to guide dosing in vulnerable populations such as children or pregnant women. However, for guidance on the use of pharmacometric methods in the registration of new drugs, both the FDA and EMA have published comprehensive documents. The Division of Pharmacometrics within the FDA’s Office of Clinical Pharmacology provides detailed guidance on the use of pharmacometrics in drug development and regulatory submissions [203]. The FDA promotes the use of MIDD, which leverages pharmacometrics for designing clinical trials, optimizing doses, and assessing E-R relationships. Specific guidelines, such as those for popPK, PBPK modeling, and E-R analyses, are included in New Drug Applications and Biologics License Applications. Moreover, the FDA has developed pharmacometric models for over a dozen diseases. However, among the 14 proposed models, there is only one model for an AID, namely, for RA [204].

The EMA has a similar set of guidelines, integrated within their Clinical Pharmacology and Pharmacokinetics sections [205]. They emphasize the importance of pharmacometric models in supporting decisions regarding the efficacy and safety of drugs, especially during the marketing authorization process. Applicants are encouraged to incorporate pharmacometric analyses when submitting new drugs for approval; however, deviations from the guidelines must be justified and discussed through scientific advice prior to submission.

While pharmacometric methods offer powerful frameworks for analyzing and predicting drug behavior, their limitations must be considered when interpreting results. Improvements in data availability, computational techniques, and model validation approaches will enhance the reliability and applicability of these methods.

## 14. Conclusions and Future Directions

There is a growing importance of pharmacometrics in the development and optimization of treatments for AIDs. Pharmacometric tools become critical in predicting drug behavior and effects across different patient populations, thus enabling more personalized treatment strategies. There are many examples available indicating that these models help to optimize dosing regimens, reduce the risk of adverse effects, and improve therapeutic outcomes. The research demonstrates that, by integrating clinical and pre-clinical molecular and animal disease model data, pharmacometrics can uncover valuable insights into disease mechanisms, treatment responses, and long-term therapeutic effects. These techniques are particularly useful in chronic and complex diseases, such as AIDs, where patient-specific factors greatly affect treatment efficacy [164,177,204].

As for future directions, the integration of AI and ML with pharmacometrics is expected to play a transformative role [183,184]. AI and ML can enhance the predictive power of these models by allowing for analyzing large datasets, identifying novel key biomarkers, and generating virtual patients for simulations [188]. This may not only refine drug development processes but also aid in designing more effective, personalized treatment plans. Furthermore, there is an ongoing effort to create more focused pharmacometric models that include only the most relevant features, reducing complexity while maintaining accuracy.

Another promising avenue involves the use of advanced modeling techniques to simulate drug interactions within complex biological systems, particularly for AIDs, where multiple drugs are often required. PBPK modeling may be especially useful in these applications; however, it is not as commonly used in studying drug–drug interactions in autoimmune patients as in the treatment of other diseases, such as malignancies [206]. These models may help clinicians to tailor treatments more precisely, minimizing harmful drug–drug interactions and optimizing therapeutic efficacy in AIDs.

There is a significant need to train new pharmacometricians, as well as research and clinical staff, to introduce them to the potential that pharmacometrics offers and to promote the widespread use of these methods not only in R&D but also in clinical practice [207]. This is especially important in low- and middle-income countries, in which, by improving treatment outcomes, maintaining remission of AIDs, and avoiding adverse reactions to treatments and exacerbations of AIDs, the application of pharmacometric methods may substantially reduce the costs of treatment, thereby increasing the availability of innovative treatments in these populations.

## Figures and Tables

**Table 1:** Examples of biomarkers used in diagnosis and monitoring of AIDs.

Biomarker	AIDs	Application	Specificity	Ref.
Rheumatoid factor (RF)	RA	Disease diagnosis	Can be present in other diseases or in healthy individuals	[34]
Anti-CCP antibody	RA	Disease classification, prognosis, and staging	High specificity	[34]
Antinuclear antibody (ANA)	SLE	Disease classification, prognosis, and staging	May also occur in other autoimmune inflammatory diseases	[33]
Anti-ssDNA antibody	SLE	Disease classification, assessment of activity	Less specific than anti-dsDNA antibody	[33]
Anti-dsDNA antibody	SLE	Disease classification, disease monitoring, particularly kidney status	Specific to SLE	[33]
Anti-Sm antibody	SLE	Disease classification, assessment of lymph node status	Specific to SLE	[33]
Anti-C1q antibodies	SLE	Disease monitoring, particularly kidney status	May also occur in other autoimmune inflammatory diseases	[33]
C3 and C4	SLE	Disease classification, monitoring of disease activity	May also occur in other autoimmune inflammatory diseases	[33]
Anti-β2GP1 antibody	APS	Disease diagnosis, risk of thrombotic complications	Low specificity	[35]
aCL antibody	APS	Disease diagnosis	Low specificity	[35]
Lupus anticoagulant (LA)	APS	Disease diagnosis, risk of thrombotic complications	High specificity	[35]
Anti-SSA and anti-SSB antibodies	SjS	Diagnosis, pregnancy complication prognosis	May also occur in other autoimmune inflammatory diseases	[36]
Anti-Scl-70 antibody	SS	Assessment of risk for complications	High specificity	[37]
Kinetochore proteins antibody (ACA)	SS	Disease diagnosis	High specificity	[38]
Anti-U1 RNP antibody	SS	Disease diagnosis	High specificity	[38]
Anti-NOR 90 antibody	SS	Disease diagnosis	High specificity	[38]
Autoantibodies to insulin (IAA)	T1D	Disease diagnosis, pathological analysis	Specific for patients not treated with exogenous insulin	[39]
Tyrosine phosphatase-like protein IA-2 (IA-2A)	T1D	Disease diagnosis, pathological analysis	High specificity	[39]
Glutamic acid decarboxylase (GADA)	T1D	Disease diagnosis, pathological analysis	May also occur in other AIDs	[39]
Zinc transporter 8 (ZnT8A)	T1D	Disease diagnosis, pathological analysis	High specificity	[39]
Anti-TSHR antibodies	GD	Disease classification, monitoring of disease activity	High specificity	[40]
Tumor necrosis factor (TNF)α	RA, AIH	Inflammatory marker	May also occur in other autoimmune inflammatory diseases	[41]
Interleukin (IL)-1β, IL-6, interferon (IFN)-γ, IL-17A	RA, AIH	Inflammatory marker	May also occur in other autoimmune inflammatory diseases	[41,42,43,44]
Alanine transaminase (ALT), aspartate transaminase (AST), γ-glutamyl transpeptidase (GGTP)	AIH, PSC, PBC	Liver damage biomarkers	Non-specific, used in various liver diseases	[41,42,43]
CRP	RA	Marker of inflammation	Non-specific, used in many inflammatory conditions	[45]
ESR (erythrocyte sedimentation rate)	RA	Marker of inflammation	Non-specific, used in various inflammatory diseases	[43]

**Table 2:** Examples of clinical outcomes used in diagnosis and monitoring of AIDs.

Abbreviation (Expansion)	AID in Which It Is Used	Short Description
Paw swelling	RA (animal models)	Measures inflammation in pre-clinical models of RA, often used to assess anti-inflammatory drug efficacy
BMD (bone mineral density)	RA, SLE	Measures bone strength, used to assess the effects of chronic inflammation and treatments on bone health
IgG (immunoglobulin G)	SLE, RA, other AIDs	Measures antibody levels in blood, indicating immune system function and autoantibody presence
SRI(4) (Systemic Lupus Erythematosus Responder Index)	SLE	Composite index assessing improvements in disease activity in SLE patients
BICLA (British Isles Lupus Assessment Group-based Composite Lupus Assessment)	SLE	Composite SLE activity measure integrating multiple organ systems
DAS28 (Disease Activity Score—28 joints)	RA	Quantifies disease activity by counting swollen/tender joints and inflammatory markers
HAQ (Health Assessment Questionnaire)	RA, SLE	Measures physical function and disability in patients
Global Pain Score	RA, SLE, other AIDs	Subjective measure of overall pain intensity
SDAI (Simplified Disease Activity Index)	RA	Composite score measuring RA disease activity
CDAI (Clinical Disease Activity Index)	RA	Clinical score measuring RA activity based on affected joint counts and other assessments
HAQ-DI (Health Assessment Questionnaire Disability Index)	RA	Detailed version of HAQ, measuring disability across multiple functional domains
ACR20/50/70 (American College of Rheumatology 20/50/70 criteria)	RA	Criteria representing 20%, 50%, or 70% improvement in RA symptoms
DAS28-CRP (Disease Activity Score (28 joints) with CRP)	RA	Variation of DAS28 using CRP levels instead of ESR
21-IFNGS (21-gene type I interferon gene signature)	SLE	Reflects activity of the type I interferon pathway, used in SLA to measure immune dysregulation

**Table 3:** Examples of pharmacometric approaches used in pre-clinical studies on AIDs.

Authors, Year, Ref.	Medication (Mechanism of Action)	Disease Studied	Modeling Approach	Main Conclusions
Earp, Dubois, Molano, Pyszczynski, Keller, et al., 2008 [44]	DEX (corticosteroid)	RA	PK/PD, DisP	The model accurately described DisP and corticosteroid effects, providing insights into optimal dosing strategies for RA treatment
Earp, Dubois, Molano, Pyszczynski, Almon, et al., 2008 [73]	DEX (corticosteroid)	RA	PK/PD, DisP	Lower doses of DEX can effectively suppress key cytokines related to bone erosion, suggesting that optimal dosing can mitigate adverse effects on BMD while controlling inflammation
Earp, Pyszczynski, et al., 2008 [70]	DEX (corticosteroid)	RA	NCA, PopPK	The study concluded that although there was a difference in clearance between healthy and arthritic rats, it was minor and unlikely to affect DEX disposition in arthritic rats
Earp et al., 2009 [71]	N/A	RA	Population DisP model	The study concluded that Dark Agouti rats may provide a more dynamic range of edema response than Lewis rats, and the onset time of the disease varied significantly between groups
Lon et al., 2011 [64]	Etanercept (TNFα inhibitor)	RA	PK/PD/DisP	Etanercept modestly reduces paw swelling in CIA rats; potential applicability to other anti-cytokine biologic agents for RA
D. Liu et al., 2011 [69]	Anakinra (IL-1 receptor antagonist)	RA	PK/PD/DisP	Anakinra had modest effects on paw edema in CIA rats, with effective modeling of PK and paw swelling data
Song and Jusko, 2011 [63]	DEX (corticosteroid)	RA	PopPK/PD/DisP	DEX effectively suppressed paw edema in both sexes; comprehensive evaluation of sex differences in PK and PD
Haselmayer et al., 2016 [77]	M2951 (BTK inhibitor)	RA, SLE	PK/PD	BTK occupancy of 60% and 80% is associated with RA and SLE progression inhibition
X. Li, DuBois, Song, et al., 2017 [74]	DEX and naproxen (corticosteroid and NSAID)	RA	PK/PD/DisP	Additive effects were observed when combining DEX and naproxen; the study showed more pronounced beneficial effects in males
Świerczek et al., 2020 [78]	Dual PDE4/7 and Lisofylline (PDE4 inhibitor)	RA	PK/PD	Comparative assessment showed potential of a new PDE7 inhibitor in the treatment of AIDs
Świerczek et al., 2021 [41]	Dual PDE4/7 inhibitor	AIH	PK/PD	Mechanistic PK/PD models were developed to assess the impact of PDE4/7 inhibition on AIH progression, confirming the importance of this mechanism for the alleviation of AIH symptoms
Świerczek, Pomierny, et al., 2022 [79]	Cilostazole, rolipram, BRL-50481 (selective PDE3, PDE4, and PDE7 inhibitors, respectively)	AIH	PK/PD	Selective PDE inhibitors were evaluated as potential medications for AIH, with rolipram being the most effective
Świerczek, Pociecha, et al., 2022 [65]	Dual PDE4/7 inhibitor	MS, RA	DisP	A novel dual PDE4/7 inhibitor showed efficacy in the inhibition of MS progression

**Table 4:** Examples of pharmacometric approaches used in translation of the results of pre-clinical studies on AIDs into clinical applications.

Authors, Year, Ref.	Medication (Mechanism of Action)	Disease Studied	Modeling Approach	Main Conclusion(s)
Zheng et al., 2011 [83]	MTRX1011A (anti-CD4 mAb, FcRn binding)	RA	PK/PD	N434H mutation did not significantly alter PK/PD relationship; challenges in translating pre-clinical findings to clinical applications due to variability
Dowty et al., 2014 [82]	Tofacitinib (JAK inhibitor)	RA	PK/PD	Efficacy of tofacitinib in RA is driven by its IC_50_; continuous daily inhibition not required for efficacy
Biliouris et al., 2018 [56]	BIIB059 (anti-BDCA2 mAb)	SLE	PK/PD and allometric scaling	Model predictions matched clinical results; this method aids in selecting safe doses for initial human trials
Wong et al., 2019 [81]	Various drugs, including indomethacin, methotrexate, etanercept, tofacitinib, and DEX	RA	PK/PD	Improved understanding of dose–efficacy relationships in pre-clinical RA models can enhance translation to clinical settings
Lledo-Garcia et al., 2022 [85]	Rozanolixizumab (anti-FcRn mAb)	Various AIDs	PopPK/PD	Model accurately predicted human responses, aiding in future clinical trial designs
Rajlic et al., 2024 [86]	mRNA-6231 (mRNA therapeutic encoding mutein IL-2)	Various AIDs	KPD	Mechanistic KPD model predicted PD response in humans, supporting dose selection for clinical trials

**Table 5:** Summary of population modeling applications in clinical settings for the treatment of AIDs.

Authors, Year, Ref.	Drug and Mechanism of Action	Disease Studied	Modeling Approach	Conclusion(s) of the Study
Yim et al., 2005 [138]	Etanercept—TNFα inhibitor	Juvenile RA	PK/PD	The popPK model accurately predicted the PK profiles of etanercept in pediatric patients, supporting the feasibility of a once-weekly dosing regimen
Frey, Grange, and Wood-worth, 2010 [107]	Tocilizumab—IL-6 receptor antagonist	RA	PK	The PK model described the PK characteristics of tocilizumab and identified key covariates, providing insights for optimizing dosing regimens in RA patients
Hu et al., 2011 [104]	Golimumab—TNFα inhibitor	RA	PK/PD	The model adequately described the PK and PD of golimumab, allowing for broader applications of indirect-response models in clinical trials
Sherwin et al., 2012 [119]	MPM—immunosuppressive	SLE	PK	The popPK model incorporated complex processes like enterohepatic recycling to aid in individualized dosing for pediatric SLE patients
Ait-Oudhia, Lowe, and Mager, 2012 [42]	Canakinumab—IL-1β inhibitor	RA	PK/PD	The model linked canakinumab and IL-1β concentrations to clinical outcomes, suggesting that canakinumab improves RA symptoms at 150 mg every 4 weeks
Levi, Grange, and Frey, 2013 [108]	Tocilizumab—IL-6 receptor antagonist	RA	PK/PD	Tocilizumab effectively reduced DAS28 scores, with a maximum reduction of 5.0 units in males and 4.6 units in females. The presence of neutralizing anti-tocilizumab antibodies did not affect the outcomes
Frey et al., 2010 [107]	Tocilizumab—IL-6 receptor antagonist	RA	PK	Optimized dosing of tocilizumab in RA requires consideration of individual patient factors to maximize therapeutic efficacy and minimize variability
Yang et al., 2015 [120]	Various immunosuppressants and immunomodulators	SLE	PK, PBPK	Individualized and tailored dosing approaches guided by PK algorithms could be safer, more effective, and cost-effective for treating SLE patients
Wojciechowski et al., 2015 [91]	Methotrexate, sulfasalazine, HCQ	RA	DisP	Population modeling can help identify patients with poor disease trajectories early, potentially leading to more effective personalized treatment strategies
Diao et al., 2016 [134]	Daclizumab—IL-2 receptor antagonist	MS	PK/PD	The model showed that daclizumab HYP effectively saturates CD25, expands CD56bright NK cells, and reduces Tregs in MS patients, helping optimize treatment strategies
Wendt et al., 2017 [151]	Insulin and glucagon; regulation of glucose levels	T1D	PK/PD	The PD model accurately simulates glucose levels, aiding in diabetes treatment strategy improvement
C. Li, Shoji, and Beebe, 2018 [114]	PF-04236921—anti-IL-6 mAb	RA, SLE, CD	PK, PK/PD	The integrated popPK and popPK/PD models can simulate PK and PD profiles under various dosing regimens and patient populations, aiding future clinical studies of anti-IL-6 mAbs
Petitcollin et al., 2018 [140]	Infliximab—TNFα inhibitor	CD	PK	The model could detect an increase in infliximab clearance, allowing early detection of immunization to infliximab, potentially improving treatment outcomes in pediatric CD patients
Berends et al., 2018 [144]	Adalimumab—TNFα inhibitor	CD	PK	The presence of anti-adalimumab antibodies significantly increased the clearance of adalimumab, and the newly developed model provided a better fit for the data compared to existing models
Bastida et al., 2019 [43]	Tocilizumab—IL-6 receptor antagonist	RA	PK/PD	The modeling approach confirmed the need for higher serum drug concentrations to normalize clinical variables compared to inflammatory markers
Xu et al., 2019 [110]	Sarilumab—IL-6 receptor antagonist	RA	PK	The model accurately described the PK of sarilumab, and no dose adjustment was required based on body weight or other demographics
Balevic et al., 2019 [137]	HCQ—immunomodulatory	Rheumatic diseases in pregnancy	PK	Pregnancy significantly increased the volume of distribution of HCQ, but it did not affect the clearance or the 24 h area under the concentration–time curve
Klünder et al., 2019 [97]	Upadacitinib—JAK1 inhibitor	RA	PK	The model was robust and could adequately describe upadacitinib plasma concentration–time profiles, aiding in optimizing dosing regimens for different patient populations
Akpalu et al., 2019 [146]	JNJ-61178104—bispecific AB targeting TNFα and IL-17A	Healthy subjects	PK	JNJ-61178104 was well tolerated, with no apparent safety concerns, and the model can be used to predict PK behavior and optimize dosing regimens by considering significant covariates like body weight and ADA status
Suleiman, Minocha, et al., 2019 [147]	Risankizumab—IL-23 inhibitor	Psoriasis	PK	The model can be used to predict risankizumab exposure in different patient populations and to optimize dosing regimens for better therapeutic outcomes in treating moderate-to-severe plaque psoriasis
Suleiman, Khatri, et al., 2019 [148]	Risankizumab—IL-23 inhibitor	Psoriasis, CD	PK	The PK of risankizumab was consistent across psoriasis and CD populations after accounting for body weight and baseline albumin differences
X. Yao et al., 2019 [121]	Teriflunomide sodium—immunomodulatory	SLE	PK	The popPK model can support Phase II clinical trial design for SLE by accurately describing teriflunomide’s PK
Xiaohui Li, Roy, and Murthy, 2019 [116]	Abatacept—T-cell co-stimulation modulator	RA	PK, PK/PD	The study concluded that abatacept’s effectiveness increases with higher steady-state trough concentrations, with a near-maximal response at 10 mg/mL, supporting the optimization of dosing regimens
Nader et al., 2020 [143]	Upadacitinib—JAK1 inhibitor	UC, atopic dermatitis (AD), RA, CD	PK	The model can be used to predict upadacitinib exposure in different patient populations, aiding in dose selection and optimizing treatment regimens for UC and AD
Scheetz et al., 2020 [122]	HCQ—immunomodulatory	SLE	PK	The model helped to predict how long blood HCQ levels would stay above a therapeutic threshold, informing dosing strategies during drug shortages to potentially reduce the risk of disease flares in SLE patients
Xiao Chen et al., 2020 [139]	Tacrolimus—immunosuppressive	LN	PK	The popPK model can help optimize tacrolimus dosing in children with SLE, ensuring better treatment outcomes and minimizing side effects
Kang et al., 2020 [113]	Adalimumab-adbm—TNFα inhibitor	RA	PK	Adalimumab-adbm is pharmacokinetically similar to Humira, and switching from Humira to adalimumab-adbm did not significantly impact adalimumab PK
Romano-Aguilar et al., 2020 [142]	MPA—immunosuppressive	LN	PK	Prednisone co-administration significantly increases the clearance of MPA, and this factor should be considered when prescribing MPA to optimize treatment for LN patients
Chan et al., 2020 [96]	Fenebrutinib—BTK inhibitor	RA	PK, E-R, MBMA	An MIDD approach with the proposed models can guide dose selection and regimen optimization for fenebrutinib
Zhou et al., 2021 [141]	Belimumab—B-lymphocyte stimulator inhibitor	SLE	PK	The PK of belimumab was adequately described by the final model, supporting its use in predicting drug exposure in Chinese pediatric patients with SLE
Toyoshima, Shibata, et al., 2021 [154]	Peficitinib—JAK inhibitor	RA	PK/PD	The models effectively described the treatment response over time, with baseline disease severity correlating with the magnitude of the treatment response, suggesting no need for dose adjustment
Toyoshima, Kaibara, et al., 2021 [95]	Peficitinib—JAK inhibitor	RA	E-R	The effects of covariates were consistent across both presented models, suggesting their potential application in the development of RA treatments
Monteleone et al., 2021 [150]	Eculizumab—complement component C5 inhibitor	MG	PK	The model supports the approved dosing regimen for eculizumab in gMG, ensuring effective and safe treatment
Chia et al., 2022 [130]	Anifrolumab—type I interferon receptor antibody	SLE	PK/PD	Higher anifrolumab exposure improves pharmacodynamic responses; the model can optimize dosing regimens
Yan et al., 2022 [136]	Inebilizumab—CD19-targeting mAb	NOSDs, SS, relapsing MS	PK	The PK of inebilizumab was well described by the proposed model, and the model can be used to predict how inebilizumab behaves in different patient populations, helping to optimize dosing regimens and improve treatment outcomes for AIDs
Almquist et al., 2022 [124]	Anifrolumab—anti-IFNAR1 mAb	SLE	PK	The model showed that the clearance rate decreased over time, supporting the recommended dosage of 300 mg every 4 weeks for sustained drug levels
Orestis Papasouliotis et al., 2022 [133]	Evobrutinib—BTK inhibitor	MS	PK, PK/PD	The model may be used in the future to simulate alternative dosing regimens and optimize treatment strategies for patients with RMS, ensuring better clinical outcomes
Acharya et al., 2023 [127]	Dapirolizumab pegol—CD40 ligand inhibitor	SLE	PK/PD	Dapirolizumab pegol increased the probability of transitioning from “Non-responder” to “Responder” status, showing a positive exposure-dependent effect
Dimelow, Gillespie, and van Maurik, 2023 [125]	Belimumab—B-lymphocyte stimulator inhibitor	SLE	PK	The study used a combination of Bayesian and maximum likelihood methods to develop and refine models predicting memory B-cell dynamics in patients with SLE
Pitsiu et al., 2023 [129]	Atacicept—B-cell stimulating factor and a proliferation-inducing ligand inhibitor	SLE	PK	The model accurately described atacicept concentrations and variability, supporting the selection of suitable doses for further clinical development
D. Chen et al., 2023 [153]	Tacrolimus—immunosuppressive	MG	PK/PD	The model aids in optimizing tacrolimus dosing regimens and personalizing treatment for different MG patient subpopulations
Morales et al., 2023 [60]	N/A—DisP model for T1D	T1D	DisP	The developed T1D DisP model accurately reflected data from natural history studies and can be used in clinical trial simulations to optimize trial design
Wojciechowski et al., 2023 [149]	Ritlecitinib—JAK inhibitor	Various AIDs	PK	The model included significant covariates affecting the clearance and absorption parameters of ritlecitinib, and it can be used to inform dosing recommendations in clinical drug development
Wojciechowski et al., 2015 [91]	Methotrexate, sulfasalazine, and hydroxychloroquine	RA	DisP	The model identified key predictors of treatment response
Chan et al., 2020 [96]	Fenebrutinib—BTK inhibitor	RA	PK, MBMA	MBMA provided valuable insights for optimizing dosing strategies
Kretsos et al., 2014 [100]	Olokizumab—mAb directly inhibiting IL-6	RA	PK/PD, Bayesian analysis	Utility of pharmacometrics to predict clinical outcomes and to aid in dose selection and trial design
Schmidt et al., 2013 [101]	Rituximab—mAb targeting CD20		MAPEL algorithm	Utility of virtual populations in understanding drug response variability and optimizing patient stratification in RA treatment
Chakraborty et al., 2012 [102]	Canakinumab—anti-IL1β mAb	RA, juvenile RA	PK/PD	The model helped to optimize dosing regimens and better understand the interplay among drug PK, signaling molecules, and clinical outcomes
Riva et al., 2023 [155]	Rituximab—CD19 B-lymphocyte depletion	Neurological AIDs	PK/PD	The model can predict CD19 B-lymphocyte depletion over time and may be useful for optimizing rituximab treatment in children.
Sokolov et al., 2023 [152]	Dapagliflozin—selective sodium–glucose co-transporter 2 (SGLT2)	T1D	PK/PD	The developed platform serves as a quantitative tool for in silico trials of combined dapagliflozin–insulin treatment
Gao et al., 2022 [131]	IL-2	SLE	PK/PD, DisP	The researchers identified an IL-2 dosage therapeutic window

**Table 6:** Examples of clinical applications of PBPK models in advancing treatment of AIDs.

Authors, Year, Ref.	Drug and Mechanism of Action	Disease Studied	Main Conclusion(s)
Machavaram et al., 2013 [158]	Simvastatin and cyclosporine—IL-6 mediated suppression of CYP3A4	RA	PBPK models can predict drug–drug interactions in RA patients with elevated IL-6 levels
Jiang et al., 2016 [157]	Sirukumab—anti-IL-6 mAb	RA	The PBPK model captured the modulation effect of IL-6 and sirukumab on CYP enzymes, potentially applicable to other cytokine-neutralizing ABs
Yellepeddi and Baker, 2019 [161]	Aspirin-triggered resolvin D1 (AT-RvD1)—anti-inflammatory	SjS	PBPK modeling can improve drug development and clinical trial design by predicting AT-RvD1 PK
Tse et al., 2020 [156]	Tofacitinib—JAK inhibitor	General analysis	PBPK model using Simcyp is reliable for predicting drug interactions and dosing special populations
Alqahtani et al., 2023 [159]	HCQ—immunomodulatory	RA, SLE	PBPK model predicted HCQ PK and supported dose adjustments in liver and kidney disease patients
Wang et al., 2022 [160]	Anti-IL-6 treatment	Various AIDs	PBPK model evaluated drug–disease interactions in inflammatory conditions
Sharma et al. [162]	Anifrolumab—mAb targeting type I IFN receptor	Various AIDs	PBPK model assesses drug tissue distribution and receptor occupancy in SLE
Effinger et al., 2021 [163]	Budesonide—corticosteroid	CD	In vitro biorelevant dissolution methodology and a PBPK model to predict the performance of budesonide in healthy subjects and CD patients

**Table 7:** Summary of MBMA applications in the treatment of AIDs.

Authors, Year, Ref.	Drug Studied and Mechanism of Action	Disease Studied	Main Conclusion(s)
Chen et al., 2021 [172]	Eculizumab—complement inhibition; Efgartigimod—FcRn antagonist	MG	Both drugs are effective in reducing MG scores; placebo effects increase significantly over 12 weeks
Chan et al., 2020 [96]	Fenebrutinib—BTK inhibitor	RA	MBMA validated clinical efficacy (ACR20/50/70), optimizing data interpretation for fenebrutinib in RA
Wang et al., 2015 [173]	Various drugs (13 classes)	RA	The 3-month ACR50 efficacy strongly predicts 6-month outcomes, supporting early efficacy-based decision-making
Demin et al., 2012 [174]	Various drugs	RA	Longitudinal MBMA enhances understanding of drug response time-course, improving indirect comparisons
Goteti et al., 2023 [175]	Various treatments	SLE	Bayesian MBMA identified dose–response and covariates, aiding MIDD for SLE
Sui et al., 2023 [176]	Ocrelizumab—anti-CD20 mAb	Primary progressive MS	Ocrelizumab demonstrated superior efficacy, offering crucial data for clinical use and trials in MS
Liu et al., 2023 [177]	Secukinumab—IL-17A inhibitor	Plaque psoriasis	MBMA identified optimal dose regimens tailored to body weight, improving cost-effectiveness and efficacy
Dodds et al., 2013 [178]	Anti-TNF, ustekinumab—anti-IL-12/23, methotrexate	Various AIDs	MBMA enhances trial design and decision-making, improving dose–response learning and Go/No-Go decisions
Maloney et al., 2024 [179]	16 drugs—various mechanisms	PsA	MBMA model predicted trial outcomes, providing insights on treatment efficacy and aiding in future trial designs
Miyano et al., 2022 [181]	Dupilumab—IL-4/IL-13 inhibitor; others	AD	MBMA simulated efficacy for nine biologics, identifying potential treatments for poor dupilumab responders
